# Towards Optimized Bioavailability of ^99m^Tc-Labeled Barbiturates for Non-invasive Imaging of Matrix Metalloproteinase Activity

**DOI:** 10.1007/s11307-021-01668-z

**Published:** 2021-11-08

**Authors:** Lisa Honold, Melanie Austrup, Andreas Faust, Christian Paul Konken, Katrin Schwegmann, Bastian Zinnhardt, Constantin Gabriel Daniliuc, Günter Haufe, Michael Schäfers, Klaus Kopka, Sven Hermann

**Affiliations:** 1 European Institute for Molecular Imaging, University of Münster, 48149 Münster, Germany; 2 Cells in Motion Interfaculty Centre, University of Münster, 48149 Münster, Germany; 3 Department of Nuclear Medicine, University Hospital Münster, 48149 Münster, Germany; 4 Department of Organic Chemistry, University of Münster, 48149 Münster, Germany; 5 Interdisciplinary Center for Clinical Research, University of Münster, 48149 Münster, Germany; 6 Institute of Radiopharmaceutical Cancer Research, Helmholtz-Zentrum Dresden-Rossendorf, 01328 Dresden, Germany; 7 Faculty of Chemistry and Food Chemistry, School of Science, Technical University Dresden, 01062 Dresden, Germany

**Keywords:** Matrix metalloproteinase inhibitors, Barbiturates, Single photon emission computed tomography, Tumor imaging, Pyrimidine-2,4,6-triones

## Abstract

**Introduction:**

Dysregulated activity of matrix metalloproteinases (MMPs) drives a variety of pathophysiological conditions. Non-invasive imaging of MMP activity *in vivo* promises diagnostic and prognostic value. However, current targeting strategies by small molecules are typically limited with respect to the bioavailability of the labeled MMP binders *in vivo*. To this end, we here introduce and compare three chemical modifications of a recently developed barbiturate-based radiotracer with respect to bioavailability and potential to image MMP activity *in vivo*.

**Methods:**

Barbiturate-based MMP inhibitors with an identical targeting unit but varying hydrophilicity were synthesized, labeled with technetium-99m, and evaluated *in vitro* and *in vivo*. Biodistribution and radiotracer elimination were determined in C57/BL6 mice by serial SPECT imaging. MMP activity was imaged in a MMP-positive subcutaneous xenograft model of human K1 papillary thyroid tumors. *In vivo* data were validated by scintillation counting, autoradiography, and MMP immunohistochemistry.

**Results:**

We prepared three new ^99m^Tc‐labeled MMP inhibitors, bearing either a glycine ([^99m^Tc]**MEA39**), lysine ([^99m^Tc]**MEA61**), or the ligand HYNIC with the ionic co-ligand TPPTS ([^99m^Tc]**MEA223**) yielding gradually increasing hydrophilicity. [^99m^Tc]**MEA39** and [^99m^Tc]**MEA61** were rapidly eliminated via hepatobiliary pathways. In contrast, [^99m^Tc]**MEA223** showed delayed *in vivo* clearance and primary renal elimination. In a thyroid tumor xenograft model, only [^99m^Tc]**MEA223** exhibited a high tumor-to-blood ratio that could easily be delineated in SPECT images.

**Conclusion:**

Introduction of HYNIC/TPPTS into the barbiturate lead structure ([^99m^Tc]**MEA223**) results in delayed renal elimination and allows non-invasive MMP imaging with high signal-to-noise ratios in a papillary thyroid tumor xenograft model.

**Supplementary Information:**

The online version contains supplementary material available at 10.1007/s11307-021-01668-z.

## Introduction

Matrix metalloproteinases (MMPs) comprise a subfamily of the metzincins and belong to the zinc‐ and calcium depending endopeptidases. MMPs are involved in a variety of physiological processes but also play a crucial role in different pathophysiological conditions, e.g., in cancer, joint disorders (including rheumatoid arthritis and osteoarthritis), neurodegenerative diseases, respiratory disorders, cardiovascular disease, and many more [1].

MMPs are capable to enzymatically cleave the protein components of the extracellular matrix (ECM) with overlapping substrate specificities. Moreover, MMPs are involved in processing bioactive molecules such as proteinase inhibitors, growth factors, cytokines, and chemokines [2, 3].

The non-invasive detection and assessment of locally upregulated and activated matrix metalloproteinases (MMPs) *in vivo* using MMP inhibitor-based radiotracers for positron emission tomography (PET) or single photon emission computed tomography (SPECT) is still a challenge [4]. However, if successful, the visualization of MMP activity by means of aforementioned scintigraphic technologies would become a breakthrough by improving diagnosis and assessment of disease progression [5].

Several groups are working on the design, improvement, and evaluation of MMP inhibitor-based radiotracers aiming at the non-invasive imaging of MMP-associated diseases by means of SPECT or PET [4, 6–12]. For this purpose, different classes of radiolabeled MMP inhibitors (MMPIs) have been developed and explored as radiotracers. Radiolabeled hydroxamate-based MMPIs have been successfully applied to image MMP activity, for example in preclinical models of atherosclerosis [13, 14] and stroke [15] and to visualize MMP activity in patients with multiple sclerosis [16]. These lead structures mainly behave like so called combined or right-hand side MMP inhibitors depending on the substituents occupying both the S1‐S3 and S1’‐S3’ enzyme pockets (combined) or S1’‐S3’ enzyme pockets (right hand side). Via such MMP inhibitor radiotracer approaches, it is putatively feasible to follow locally upregulated MMPs in their activated forms *in vivo* [5, 13, 17–20]. Disadvantages of hydroxamates in MMP imaging are their broad inhibition spectrum, metabolic instability, as well as interactions with other metalloproteinases due to their high transition-metal binding potential [21]. Non-hydroxamate-based MMPIs like substituted pyrimidine-2,4,6-triones (barbiturates) often possess higher specificity for the gelatinases MMP-2 and MMP-9 [22] and were the basis for the development of C-5-disubstituted barbiturates with improved MMP specificity and potency [23]. We have introduced ^18^F-labeled C5‐disubstituted barbiturates as potential MMP‐targeted radiotracers, putatively binding to the zinc ion at the active site via the enolic tautomer of the barbiturate moiety [24–26]. Moreover, we have also presented a first ^68^Ga‐labeled version of a barbiturate which was synthesized by azide‐alkyne cycloaddition. This potential PET tracer was the first radiometal-labeled MMP inhibitor based on a barbiturate lead structure reported so far [27]. All these examples are characterized by fast blood clearance and therefore short availability for binding to the target enzymes which is mainly caused by their pharmacokinetic profile rather than by their binding properties to the active MMPs. Introduction of ionic charges like in [^99m^Tc]**RP805,** a cyclic hydroxamate-based MMP-radiotracer, gave promising results in imaging of MMP activation [28].

Therefore, we developed and evaluated three MMPIs presenting a novel series of ^99m^Tc‐labeled barbiturates with gradually increasing hydrophilicity. Starting with a glycine-spacer ([^99m^Tc]**MEA39**), we increased hydrophilicity by changing the spacer to lysine ([^99m^Tc]**MEA61**) and finally introduced HYNIC as a bifunctional coupling agent for ^99m^Tc-labeling, together with TPPTS and tricine as co-ligands, resulting in high hydrophilicity with regard to the final radiotracer ([^99m^Tc]**MEA223**). We evaluated the impact of altered MMPI hydrophilicity on tracer dynamics and clearance in wild-type mice *in vivo*. Furthermore, we investigated the potential of these ^99m^Tc‐labeled barbiturates to non-invasively assess tumor-associated MMP activity, specifically whether the altered tracer hydrophilicity and dynamics leads to improved tumor/blood contrast.

## Materials and Methods

### Chemistry

All chemicals, reagents, and solvents for the syntheses of the compounds were analytical grade, purchased from commercial sources and used without further purification unless otherwise specified. For radiosynthesis, only solvents of pharmaceutical purity (Reag.Ph.Eur.) were used.

The labeling of compounds was accomplished by using two different kits which both are prepared in house. Briefly, one labeling kit to produce a triaquatricarbonyl-complex [^99m^Tc(CO)_3_(OH_2_)_3_]^+^ was used for radiosynthesis of [^99m^Tc]**MEA39** and [^99m^Tc]**MEA61**; the second one was used for the HYNIC-derivative [^99m^Tc]**MEA223** (see SI). The precursors and corresponding kits were heated to 100 °C for 16–20 min. After cooling to room temperature, the crude reaction mixtures were purified by HPLC. Products were collected, diluted with water, and filtered through Sep-Pak® C-18 Plus cartridges. Solvents were removed under reduced pressure without heating, and the residues were solved in 0.9% NaCl-solution containing 1.6 vol% of Tween80® (100–500 µL). For quality control, all injectable solutions were analyzed determining the radiochemical purity (RCP), the pH-value, and osmolality.

Identification of labeled compounds was performed by co-injection of a non-radioactive Rhenium-based reference on HPLC. In the case of [^99m^Tc]**MEA223,** a reference compound could not be synthesized, and the identification of the corresponding ^99g^Tc-derivative (after decay) was done by mass spectrometry.

The serum stability of all radiolabeled compounds was evaluated by incubation in human and murine serum at 37 °C for up to 120 min and analyzed by HPLC. The distribution coefficients *(logD*_*exp*_*)* were determined in a two-phase system consisting of 1-octanol and PBS-buffer (pH = 7.4) according to the literature [29].

### Animals

All animal experiments performed in the study were in accordance with the German Law on the Care and Use of Laboratory Animals and approved by the local authorizing agency of North Rhine-Westphalia.

C57/BL6 mice (female, 12–15 weeks, 20–23 g) were anesthetized with 2% isoflurane (Abbott Animal Health) in 100% O_2_, and a lateral tail vein catheter was placed using a 27G needle connected to 15-cm polyethylene tubing. 80–100 MBq of the respective tracer was injected as a bolus (100 μL compound flushed with 100 μL saline) via the tail vein, and subsequent SPECT imaging was performed.

For tumor studies, 2 × 10^6^ K1-LITG human thyroid cancer cells in 40–60 µl plain DMEM medium (Thermo Fisher Scientific, Waltham, USA) were subcutaneously injected above each shoulder of CD1^nude/nude^ mice (Charles River, female, 9–10 weeks, 25–28 g). Imaging experiments were performed 15 days post-implantation. All animals were randomly assigned to experimental groups.

### SPECT/CT Imaging

SPECT experiments were carried out using a small-animal SPECT/CT scanner (NanoScan, Mediso). For biodistribution studies, dynamic SPECT scans were acquired over the course of 90 min p.i. (9 × 10 min frames, field of view 108 mm). Following the acquisition, CT contrast agent (Ultravist^®^-370, 5 µl/g bw) was injected via the tail vein catheter, and a CT image was obtained. Mice underwent subsequent SPECT/CT scans 4 h p.i. (1 × 30 min frame) and 24 h p.i. (1 × 60 min frame). For *in vivo* tumor uptake studies, mice were imaged 0–60 min and 4 h p.i. of tracer with a reduced field of view (1 × 30 min frame, 26 mm).

Reconstructed image data sets were analyzed using the in-house developed software MEDgical. Three-dimensional volumes of interest (VOIs) were defined over the respective organs in CT data sets, transferred to the co-registered SPECT data, and analyzed quantitatively. Regional uptake was calculated as percentage injected dose (%ID) or standardized uptake units (SUV) by decay-correcting all data to the time of injection and subsequently dividing counts per milliliter in the VOI by total injected dose and normalizing to bodyweight. Total elimination was analyzed by thresholding the abdominal region in dynamic SPECT acquisitions. Renal elimination was defined as the sum of radioactivity in kidneys and urinary bladder, hepatobiliary elimination as the difference between total elimination and renal elimination.

### *Ex Vivo *Validation

Following the last SPECT/CT acquisition, mice were euthanized by cervical dislocation and a necropsy was performed. Ex vivo biodistribution of radioactivity was analyzed by scintillation counting (Wizard2 gamma counter, Perkin Elmer), and the radioactivity in respective organs was decay-corrected and calculated as %ID per Gram tissue (% ID/g).

Directly after scintillation counting, tumors were embedded and snap-frozen in Tissue-Tek (Tissue-Tek OCT Weckert Labortechnik, Kitzingen, Germany). For autoradiography, 20-µm frozen tissue sections were measured for 6 h in a microimager (Biospace Lab, Nesles la Vallee, France). Adjacent Sects. (10 µm) were collected for histological analysis and stained with an anti-MMP-9 antibody (abcam ab38889, 1:200, overnight at 4 °C), the appropriate secondary antibody (anti-rabbit A-21206, Invitrogen, 1:800) and DAPI (Vectashield, H-1500, Vector Laboratories, USA).

### Statistical Analysis

Statistical significance was analyzed using 1-way or 2-way ANOVA and Tukey post-tests. *P* values of *p < 0.05, **p < 0.01, and ***p < 0.001 were considered significant. In-text values are mentioned as mean ± standard deviation.

## Results

**Scheme 1.**
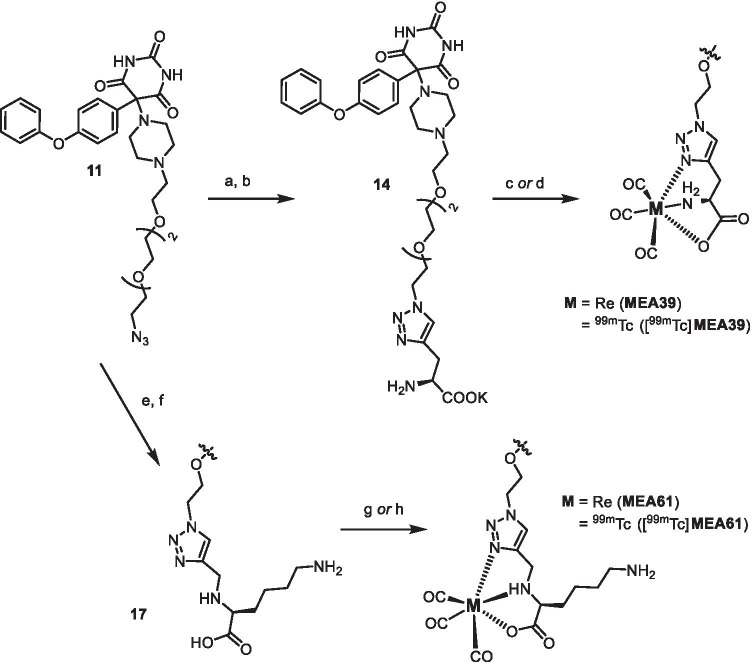
Synthesis and radiolabeling of [^99m^Tc]**MEA39** and [^99m^Tc]**MEA61**: (a) (*S*)-2-[(*tert*-butoxycarbonyl)amino]pent-4-ynoic acid, CuSO_4_ · 5H_2_O, sodium ascorbate, DMF, H_2_O, rt, 12 h, 40%; (b) (I) TFA, CH_2_Cl_2_, rt, 18 h, (II) K_2_CO_3_, THF, rt, 5 h, 16% (two steps); (c) [NEt_4_]_2_[ReBr_3_(CO)_3_], MeOH, H_2_O, 70 °C, 5 h, then rt, 18 h, 18%; (d) [^99m^Tc(CO)_3_(OH_2_)_3_]^+^, 100 °C, 16 min., rcy 21% d.c., rcp > 99% (e) **8**, CuSO_4_ · 5H_2_O, Na ascorbate, DMF, H_2_O, rt, 12 h, 48%; (f) (I) TFA, CH_2_Cl_2_, rt, 18 h, 63% (II) K_2_CO_3_, THF, rt, 5 h, 79%; (g) [NEt_4_]_2_[ReBr_3_(CO)_3_], MeOH, H_2_O, 70 °C, 5 h, then rt, 18 h, 41%; (h) [^99m^Tc(CO)_3_(OH_2_)_3_]^+^, 100 °C, 16 min., rcy 23% d.c., rcp > 99%. (d.c.: decay corrected; rcy: radiochemical yield; rcp: radiochemical purity).

The synthesis of the three different ^99m^Tc-labeled MMPIs starts with the preparation of the barbiturate **11** with an azido PEG-chain for installing the different Tc-chelators in all cases [30]. This azide-functionalized derivative was also characterized by crystal structure which is outlined together with the synthetic procedures in the supporting information. For the first two ^99m^Tc-labeled tracers [^99m^Tc]**MEA39** and [^99m^Tc]**MEA61,** we applied the “click to chelate” concept [31]. In contrast to the literature, the corresponding precursors for labeling which are usually prepared in situ were isolated because direct labeling failed. We assume that this is likely being caused by the two very basic tertiary amines of the piperazine ring interfering with the amino acid. This makes an in situ formation of the necessary precursors impossible or at least unfavorable. For [^99m^Tc]**MEA39,** the azide **11** was used in a click reaction with the commercially available fully functionalized (*S*)-2-[(*tert*-butoxycarbonyl)amino]pent-4-ynoic acid to get the protected precursor **13**. After deprotection and purification (as potassium salt), the corresponding Re-complex **MEA39** was prepared as cold reference using [NEt_4_]_2_[ReBr_3_(CO)_3_][32] (Scheme 1). For radiolabeling an in-house prepared [^99m^Tc(CO)_3_(OH_2_)_3_]^+^-kit was used yielding [^99m^Tc]**MEA39** with a radiochemical yield of 21 ± 11% (*n* = 6) and reproducible radiochemical purity of over 99%. The total time for synthesis was 131 min ± 9 min (*n* = 6). The identity of the product was verified by HPLC by co-injection of the analogous Rhenium-complex **MEA39** as well as by mass spectrometry. For [^99m^Tc]**MEA61,** we installed the more polar lysine-derivative **8** which was prepared according to the literature [33]. After forming the triazole and subsequent deprotection, the resulting precursor **17** was converted into the corresponding rhenium complex (**MEA61**) and radiolabeled with technetium-99 m ([^99m^Tc]**MEA61)** in the same way as mentioned before. The radiochemical yield was comparable with 23 ± 11% (*n* = 3) and radiochemical purities of over 99% (130 min total synthesis time).

**Scheme 2.**
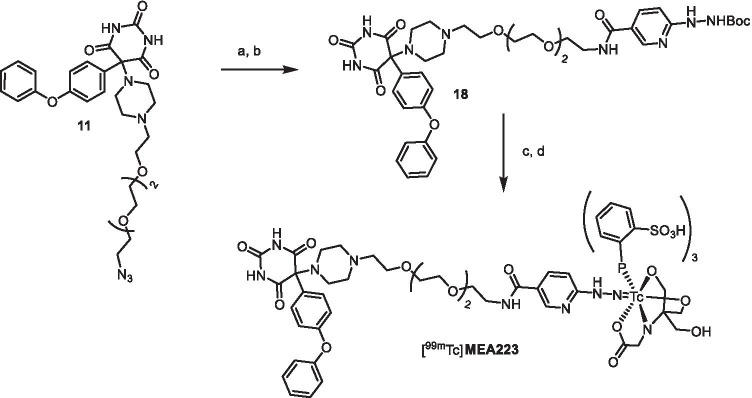
Synthesis and radiolabeling of [^99m^Tc]**MEA223**: (a) PPh_3_, THF, H_2_O, rt, 2d, 58%; (b) 2,5-Dioxopyrrolidin-1-yl-6-[2-(*tert*-butoxycarbonyl)hydrazinyl]nicotinate, THF, NEt_3_, rt, 12 h, 79%; (c) HCl, dioxane, MeOH, THF, 4d, 18% (d) [^99m^TcO_4_]^−^, 3,3′,3″-phosphanetriyltris-(benzenesulfonic acid) trisodium salt (TPPTS), tricine, mannitol, disodium succinate hexahydrate, succinic acid, 100 °C, 20 min., rcy 34% d.c., rcp > 99%. (d.c.: decay corrected; rcy: radiochemical yield; rcp: radiochemical purity).

In order to further improve solubility and hydrophilicity of the envisioned tracers, a HYNIC-functionalized barbiturate was prepared. After Staudinger-reduction of the azide **11** to the free amine, the active ester 2,5-dioxopyrrolidin-1-yl-6-[2-(*tert*-butoxycarbonyl)-hydrazinyl]nicotinate was used to form the amide **18**. The Boc-group was removed with hydrochloric acid in dioxane yielding the precursor **19**. For radiolabeling with technetium-99 m, suitable co-ligands are necessary to get tracers of reasonable stability. To reach high hydrophilicity, we choose tricine and triphenylphosphine trisulfonate (TPPTS) as co-ligands and synthesized [^99m^Tc]**MEA223** with a radiochemical yield of 34 ± 10% (*n* = 10) and reproducible radiochemical purity of over 99% via standard kit-procedure (Scheme 2) [34]. The total time for synthesis was 143 min ± 30 min (*n* = 10). Under these conditions, the synthesis of the corresponding rhenium complex failed. It is known that rhenium-labeled HYNIC-biomolecule conjugates are often lacking stability [35, 36]. Therefore we performed mass spectrometry to verify [^99g^Tc]**MEA223** (see SI).

## *In vitro *Characterization

In order to evaluate the affinity of the new barbiturate-based compounds towards MMPs, an *in vitro* inhibition study was performed. The IC_50_-values were determined for MMP-2, MMP-8, MMP-9, MMP-13, and MMP-15 using the protocol from Huang et al. [37] The rhenium-labeled non-radioactive counterparts **MEA39** and **MEA61** were used in this assay, and the results are summarized in Table 1. Additionally, the corresponding precursors were also measured and outlined in the SI for comparison. For **[**^**99m**^**Tc]MEA223** the determination of IC_50_-values was not possible because of lacking the Re-labeled non-radioactive derivative (see above).

**Table 1.** MMP inhibitory activity and *Log*D.

	IC_50_ (nM) Non-radioactive Re-complex					Log*D* (compound labeled by [^99m^Tc])
	MMP-2	MMP-8	MMP-9	MMP-13	MMP-14	
**MEA39**	2.0 ± 0.5	28.0 ± 7.0	0.9 ± 0.1	2.0 ± 0.3	5.9 ± 0.7	0.79 ± 0.03
**MEA61**	3.6 ± 0.7	5.5 ± 0.4	8.0 ± 0.5	26.0 ± 0.6	14.0 ± 0.8	0.35 ± 0.23
**MEA223**	Not available	Not available	Not available	Not available	Not available	0.15 ± 0.07

The amino acid-based barbiturates **MEA39** and **MEA61** show high affinity towards the tested MMPs in the nanomolar range. The glycine-based **MEA39** has some specificity towards the gelatinases MMP-2 and MMP-9 over MMP-8, while the lysine-based **MEA61** shows selectivity for the gelatinases over MMP-13 and MMP-14.

Experimental log*D* value were determined [29] and showed decreasing lipophilicity from [^99m^Tc]**MEA39** (0.79 ± 0.03) to [^99m^Tc]**MEA61** (0.35 ± 0.23) and [^99m^Tc]**MEA223** (0.15 ± 0.07). *in vitro* stability tests were performed by incubating tracers in human and murine blood serum followed by HPLC analysis for 2 h. All tracers proved to be stable with no detectable radiometabolites or decomposition products in both human and murine serum after 120 min. (see SI).

### Preclinical Evaluation

#### Biodistribution

**Fig. 1.**
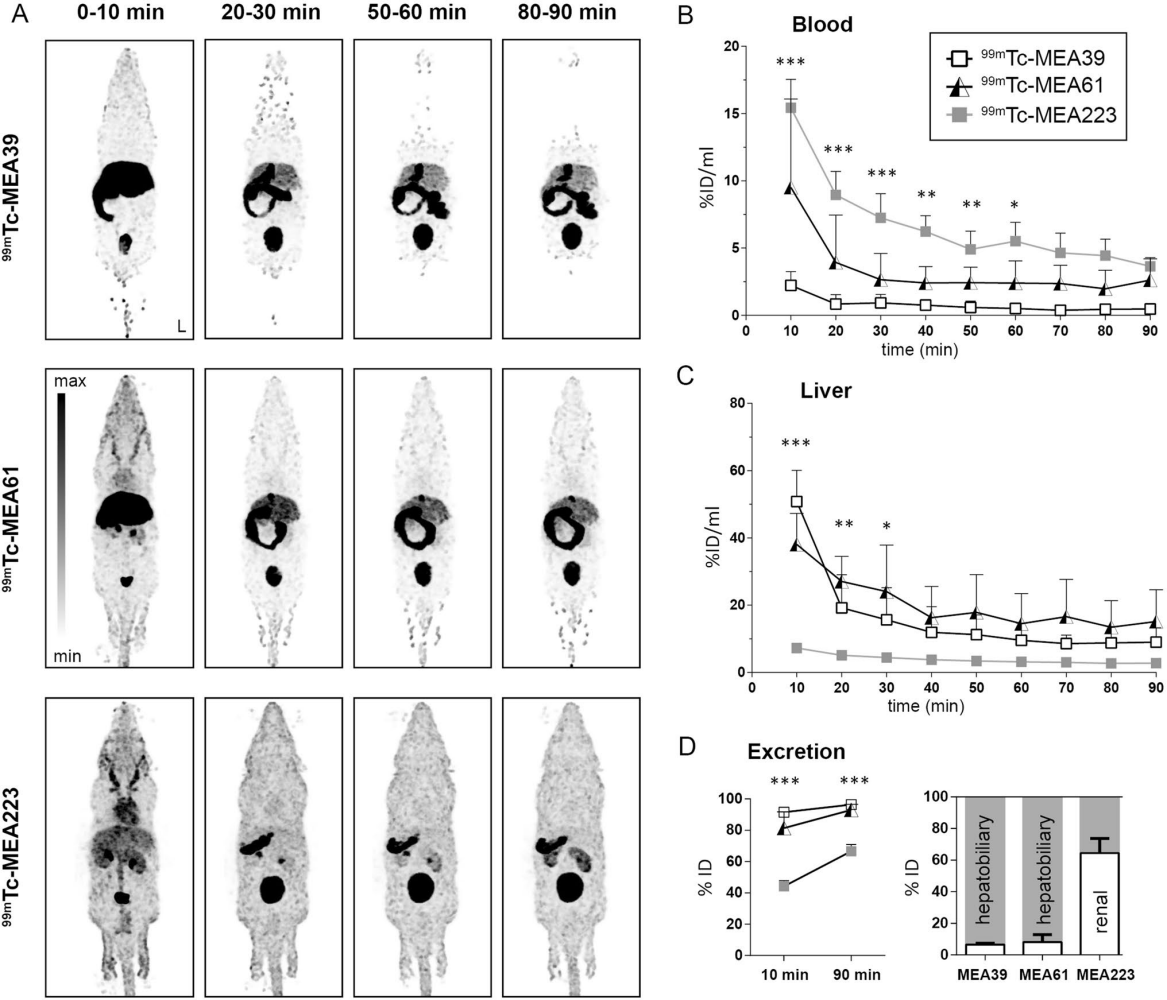
*In vivo* distribution and excretion analysis of barbiturates in adult C57/Bl6 mice after intravenous injection. **A** Maximum intensity projections of the biodistribution of radiotracers [^99m^Tc]**MEA39**, [^99m^Tc]**MEA61**, and [^99m^Tc]**MEA223** at increasing time points post injection. **B** Dynamic *in vivo* blood radioactivity determined from left ventricular volume of interest (VOI). **C** *in vivo* radiotracer accumulation in the liver. **D** Radioactivity accumulation (expressed as percentage of injected dose) in excretion organs (liver, gallbladder, intestine, kidney, bladder) at 10 and 90 min p.i. and relative percentage of hepatobiliary and renal elimination pathways. Data are shown as mean ± SD (*n* = 4–5), ID, injected dose; image orientation L, left. Statistical significance was calculated using 2-way ANOVA and Tukey post-test, and stars indicate significance of differences of radiotracer uptake of [^99m^Tc]**MEA223** compared to the other compounds: *p < 0.05, **p < 0.01, ***p < 0.001.

*In vivo* biodistribution was determined in adult female C57/BL6 mice after intravenous injection. Representative whole-body images (maximum intensity projections) of investigated radiotracers at 0–10, 20–30, 50–60, and 80–90 min post injection (p.i.) are shown in Fig. 1A.

[^99m^Tc]**MEA39** presented with a fast clearance from the blood with 91.5 ± 3.2% of the injected dose (%ID) accumulating in the excretion organs liver and kidney already within the first 10 min (Fig. 1B, C). After 90 min p.i., 96.4 ± 2.0%ID were excreted, the vast majority via the hepatobiliary system (Fig. 1D). Elimination of [^99m^Tc]**MEA61** was equally fast, showing 93.0 ± 3.6%ID accumulating in excretion organs after 90 min p.i.. Similar to [^99m^Tc]**MEA39**, the majority of [^99m^Tc]**MEA61** (92.0%) was eliminated via the hepatobiliary pathway.

In contrast, [^99m^Tc]**MEA223** showed a delayed elimination with only 66.6 ± 4.4%ID found in excretion organs 90 min p.i.. First pass effect and early accumulation in the liver was significantly reduced ([%ID/ml] at 10 min p.i.: 7.3 ± 0.5 vs. 38.1 ± 9.3 ([^99m^Tc]**MEA61)** vs. 50.8 ± 9.3 ([^99m^Tc]**MEA39**) (Fig. 1C), and elimination was shifted towards renal elimination (64.4 ± 9.3%). Until 60 min p.i., [^99m^Tc]**MEA223** showed significantly higher radioactivity in the blood than [^99m^Tc]**MEA39** and [^99m^Tc]**MEA61** (Fig. 1B).

**Fig. 2.**
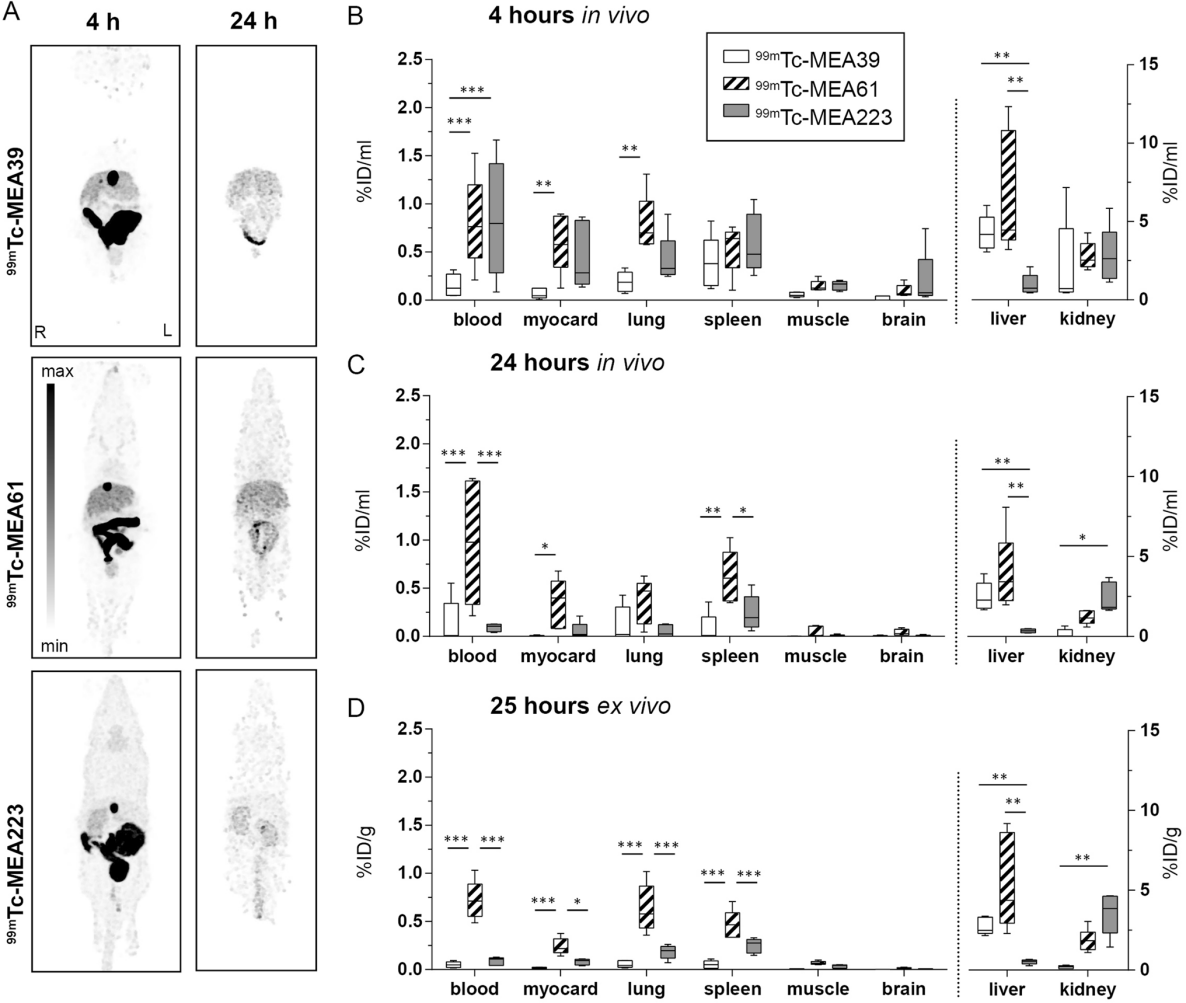
Late time point *in vivo* biodistribution of barbiturates in adult C57/Bl6 mice. Maximum intensity projections **A** and quantification **B**, **C** of the biodistribution of radiotracers [^99m^Tc]**MEA39**, [^99m^Tc]**MEA61**, and [^99m^Tc]**MEA223** 4 and 24 h p.i. **D** Ex vivo biodistribution determined by scintillation counting 25 h p.i. Data shown as box plot min to max (*n* = 5–6). Statistical significance was calculated using 2-way ANOVA and Tukey post-test: *p < 0.05, **p < 0.01, ***p < 0.001.

Additionally, late time point acquisitions were performed 4 h and 24 h after tracer injection to assess delayed kinetics. Representative examples are shown in Fig. 2. Four hours p.i. blood radioactivity of [^99m^Tc]**MEA223** and [^99m^Tc]**MEA61** remained considerably high ([%ID/ml]: 0.8 ± 0.6 and 0.8 ± 0.5), while [^99m^Tc]**MEA39** showed an increased wash out and lower blood concentrations ([%ID/ml]: 0.2 ± 0.1) as shown in Fig. 2B and C. While the blood radioactivity of [^99m^Tc]**MEA223** decreased further over the next 20 h ([%ID/ml] at 24 h p.i.: 0.1 ± 0.1), [^99m^Tc]**MEA61** presented with a long circulation time and unchanged blood radioactivity ([%ID/ml] at 24 h p.i.: 1.0 ± 0.7). Radiotracer accumulation in non-excretion organs 4 and 24 h p.i. was very low for all compounds with < 1% ID/ml, but, due to increased circulation times, as a tendency slightly higher for [^99m^Tc]**MEA61**. Complementary ex vivo measurements of tissue samples at 25 h p.i. confirmed the *in vivo* data at the latest time point (24 h p.i.) as shown in Fig. 2D.

#### *In vivo* Imaging of Tumor MMP Activity

We applied the three radiotracers for *in vivo* imaging of MMP activity in a subcutaneous xenograft model of human K1 papillary thyroid tumors which are known for high MMP activity. MMP-2 and MMP-9 activity in patients with papillary thyroid tumors are linked to tumor cell invasion and metastasis, and high gelatinase activity has been associated with poor prognosis [38, 39].

**Fig. 3.**
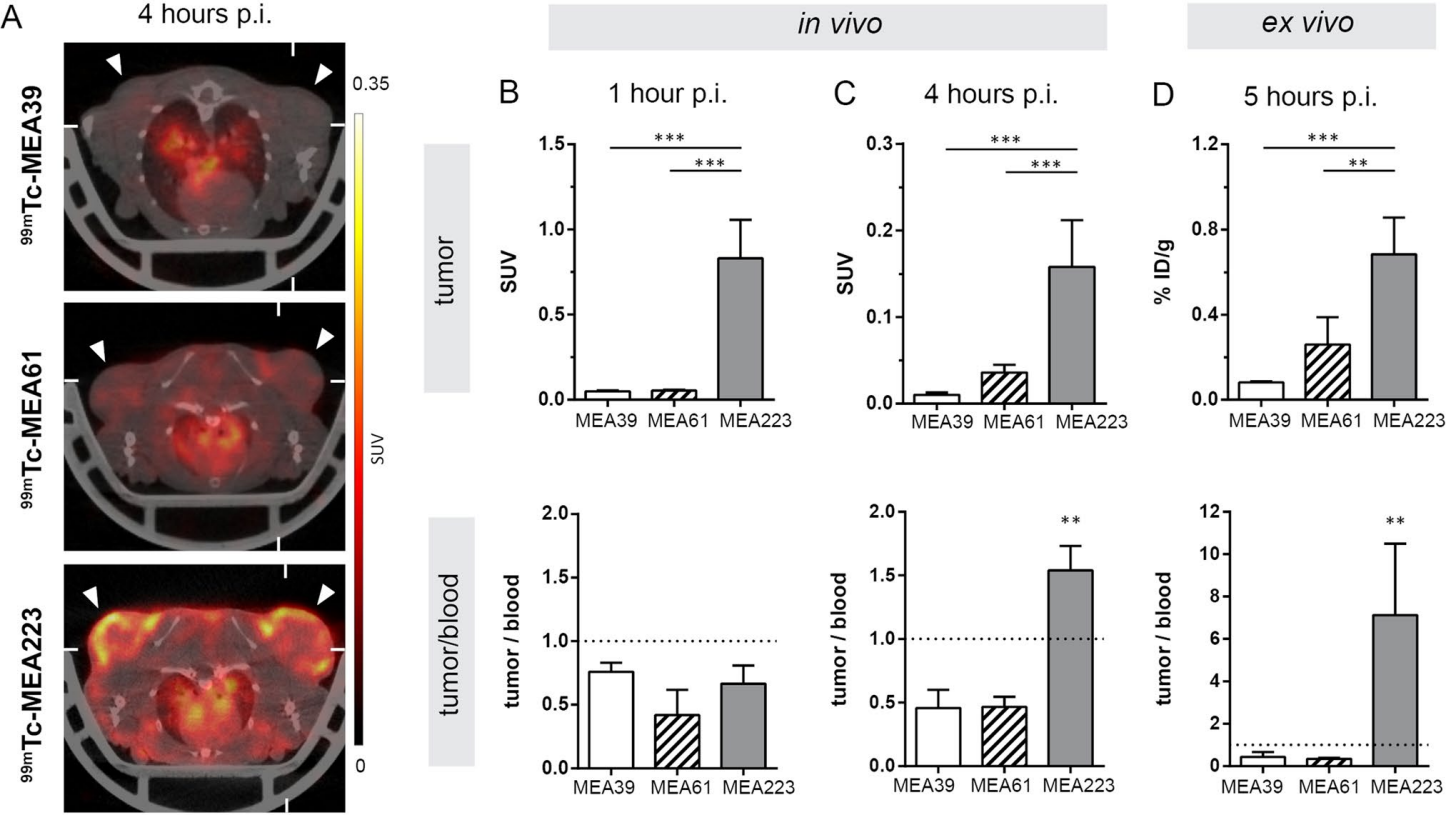
*In vivo* imaging of MMP activity in a subcutaneous xenograft tumor model. **A** Axial SPECT images coregistered to CT showing *in vivo* tumor signals 4 h post radiotracer injection (error heads point at s.c. tumors). **B**, **C** Quantification of *in vivo* tumor signals and tumor-to- blood ratios 1 h and 4 h p.i., respectively. **D** Ex vivo tumor signals determined by scintillation counting and tumor-to-blood ratios 5 h p.i. Data shown as mean ± SD (*n* = 5). Image orientation L, left; SUV, standardized uptake units; %ID/g, % injected dose per gram. Statistical significance was calculated using 1-way ANOVA and Tukey post-tests: *p < 0.05, **p < 0.01, ***p < 0.001.

Representative SPECT images (axial sections) of radiotracer uptake at 4 h p.i. coregistered to a CT are shown in Fig. 3A.

**Fig. 4.**
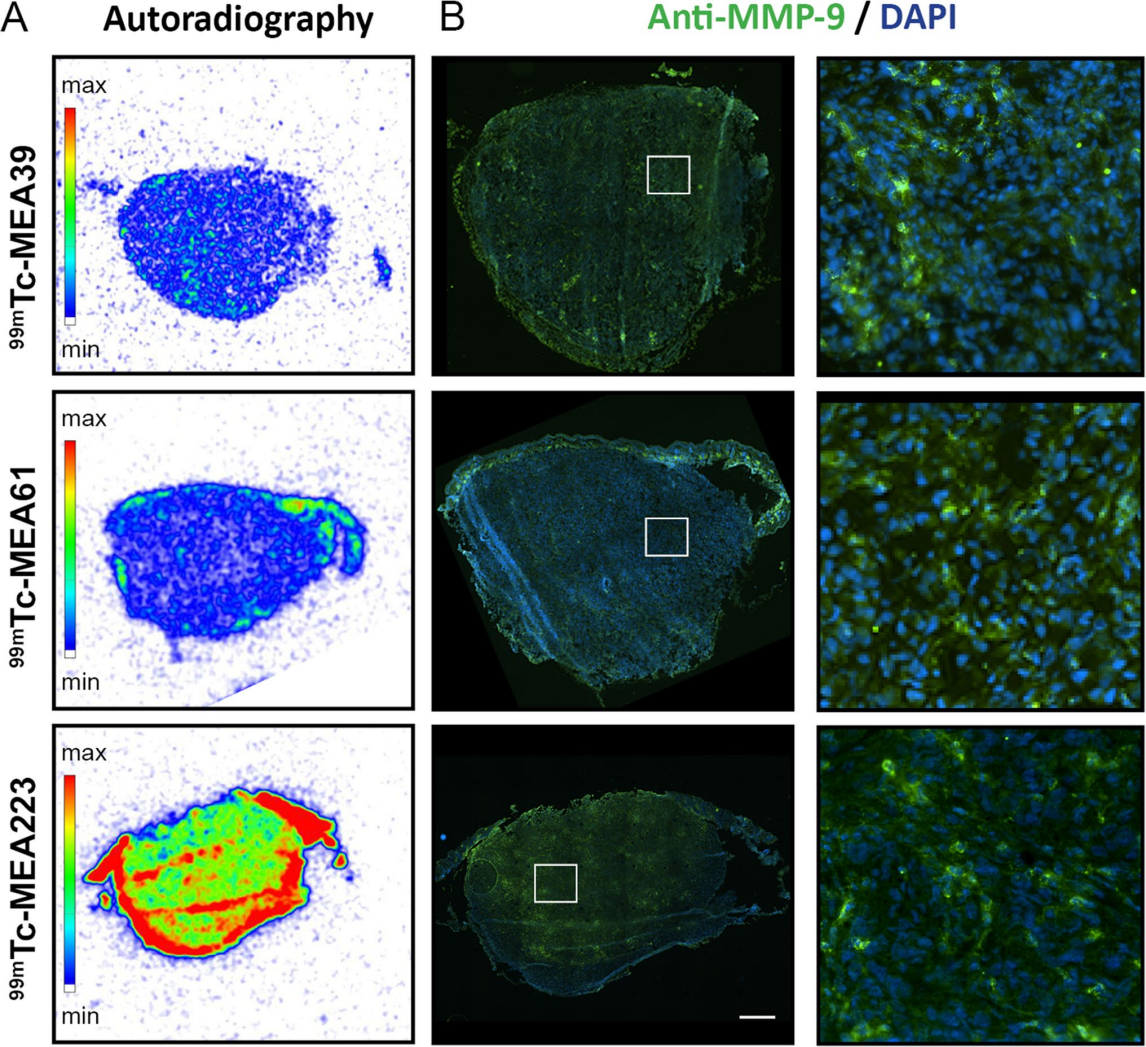
Ex vivo validation of tumor radiotracer accumulation. **A** Representative autoradiographic images of axial tumor sections, acquired 6 h post radiotracer injection. **B** Consecutive histological sections stained for MMP-9 (green) and counterstained with DAPI (blue). Scale bar represents 1 mm.

1 h p.i., *in vivo* tumor signal was highest for [^99m^Tc]**MEA223**, followed by [^99m^Tc]**MEA61** and [^99m^Tc]**MEA39**. Differences in early tumor signals were in accordance with differences in blood radioactivity, as tumor-to-blood ratios remained below 1 for all radiotracers (Fig. 3B). 4 h p.i., [^99m^Tc]**MEA223** showed a strong accumulation in tumors, independent of local tumor perfusion with positive tumor-to-blood ratios. In contrast, **[**^**99m**^**Tc]MEA61** and [^99m^Tc]**MEA39** showed no specific tumor accumulation, as tumor signals declined parallel to the decrease in blood radioactivity ([tumor/blood *in vivo* 4 h p.i.]: 1.54 ± 0.19 ([^99m^Tc]**MEA223**) vs. 0.47 ± 0.08 ([^99m^Tc]**MEA61**) vs. 0.46 ± 0.14 ([^99m^Tc]**MEA39**) (Fig. 3C). *In vivo* results could be confirmed by ex vivo scintillation counting ([tumor/blood *ex vivo* 5 h p.i.]: 7.12 ± 3.37 ([^99m^Tc]**MEA223**) vs. 0.44 ± 0.19 ([^99m^Tc]**MEA61**) vs. 0.49 ± 0.22 ([^99m^Tc]**MEA39**) (Fig. 3D). Additionally, ex vivo autoradiography confirmed that only [^99m^Tc]**MEA223** showed relevant radiotracer accumulation throughout the tumor, even though all tumors were highly positive for the target in MMP immunohistochemistry (Fig. 4).

## Conclusion

We successfully prepared two new [^99m^Tc]Tc(CO)_3_‐labeled MMP inhibitors, bearing either a glycine or a lysine residue within the ^99m^Tc‐chelator. In a third compound, HYNIC was used together with TPPTS and tricine as co-ligands. All tracers were prepared with high radiochemical purities and reproducible radiochemical yields. *In vitro* experiments showed high stability of all tracers in human and murine blood serum, and excellent affinity of the amino acid-based reference compounds **MEA39** and **MEA61** towards targeted matrix metalloproteinases. As expected, HYNIC-based [^99m^Tc]**MEA223** showed increased hydrophilicity, as compared to [^99m^Tc]**MEA39** and [^99m^Tc]**MEA61**. *In vivo*, amino acid-based tracers [^99m^Tc]**MEA39** and [^99m^Tc]**MEA61** were rapidly eliminated via hepatobiliary pathways. In contrast, the more hydrophilic [^99m^Tc]**MEA223** was primarily excreted via the kidneys and showed a significantly increased bioavailability for the first 90 min after injection. We demonstrated the relevance and impact of the altered tracer kinetics in a thyroid tumor xenograft model. Here, [^99m^Tc]**MEA223** exhibited a high tumor-to-blood ratio that could easily be delineated in SPECT images. The newly developed [^99m^Tc]**MEA223** hence allows non-invasive imaging of MMP activity with high signal-to-noise and should be investigated in additional pathophysiological conditions.

## Electronic supplementary material

Below is the link to the electronic supplementary material.

ESM 1 Supporting information is provided including detailed procedures for all synthetic steps, analytical data and spectra copies of all new compounds, western blot data, as well as detailed information about radiosynthesis and animal experiments. CCDC 2,090,342 for compound **11** is contained in the supplementary crystallographic data for this manuscript. This data can be obtained free of charge from The Cambridge Crystallographic Data Centre via http://www.ccdc.cam.ac.uk/data_request/cif. (DOCX 4084 kb)
