# Supplementary material for: Towards Optimized Bioavailability of 99mTc-Labeled Barbiturates for Non-invasive Imaging of Matrix Metalloproteinase Activity
Source: Mol Imaging Biol. 2021 Nov 8;24(3):434–43. doi: 10.1007/s11307-021-01668-z (PMC9085681; doi:10.1007/s11307-021-01668-z)
Supplement: Supplementary file 1 — Supporting information is provided including detailed procedures for all synthetic steps, analytical data and spectra copies of all new compounds, western blot data, as well as detailed information about radiosynthesis and animal experiments. CCDC 2,090,342 for compound 11 is contained in the supplementary crystallographic data for this manuscript. This data can be obtained free of charge from The Cambridge Crystallographic Data Centre via http://www.ccdc.cam.ac.uk/data_request/cif. (DOCX 4084 kb) [file 11307_2021_1668_MOESM1_ESM.docx]

**Supporting Information**

**Towards optimized bioavailability of ^99m^Tc-labeled barbiturates for non-invasive imaging of matrix metalloproteinase activity**

Honold, Lisa^1,2*^; Austrup, Melanie^3*^; Faust, Andreas^1,2,3^; Konken, Christian Paul^3^; Schwegmann, Katrin^3^; Zinnhardt, Bastian^1,3^; Daniliuc, Constantin Gabriel^4^; Haufe, Günter^4^; Schäfers, Michael^1,2,3,5^; Kopka, Klaus^6,7^; Hermann, Sven^1,2,5,A^.

*^1^ European Institute for Molecular Imaging, University of Münster, 48149 Münster, Germany*

*^2^ Cells in Motion Interfaculty Centre, University of Münster, 48149 Münster, Germany*

*^3^ Department of Nuclear Medicine, University Hospital Münster, 48149 Münster, Germany*

*^4^ Department of Organic Chemistry, University of Münster, 48149 Münster, Germany*

*^5^ Interdisciplinary Center for Clinical Research, University of Münster, 48149 Münster, Germany*

*^6^ Institute of Radiopharmaceutical Cancer Research, Helmholtz-Center Dresden-Rossendorf, 01328 Dresden, Germany*

*^7^ Faculty of Chemistry and Food Chemistry, School of Science, Technical University Dresden, 01062 Dresden, Germany*

** both authors contributed equally*

*^A^ Corresponding author, please address correspondence to “shermann@uni-muenster.de”*

Content

[1. Experimental procedures 3](#_Toc82002293)

[1.1. General considerations 3](#_Toc82002294)

[1.2. Synthetic Organic Chemistry / Spectra of new compounds 6](#_Toc82002295)

[1.2.1. Synthesis of spacer units and general side chain building blocks 6](#_Toc82002296)

[1.2.2. Synthesis of central pyrimidine-2,4,6-trione building blocks 9](#_Toc82002297)

[1.2.3. General procedure A: CuAAC of alkines with barbiturate azide (11). 14](#_Toc82002298)

[1.2.4. General procedure B: Removal of Boc protecting groups. 14](#_Toc82002299)

[1.2.5. General procedure C: Synthesis of rhenium complexes. 14](#_Toc82002300)

[1.2.6. Synthesis and spectroscopic data of the glycine based barbiturate 15](#_Toc82002301)

[1.2.7. Synthesis and spectroscopic data of the lysine based barbiturate 20](#_Toc82002302)

[1.2.8. Synthesis and spectroscopic data of the HYNIC precursor (19) 25](#_Toc82002303)

[1.3. Radiosynthesis, in vitro data 29](#_Toc82002304)

[1.3.1. General considerations 29](#_Toc82002305)

[1.3.2. ^99m^Tc-labeling using „kits“ 31](#_Toc82002306)

[1.3.3. General procedure AR for the ^99m^Tc-labeling of barbiturates 31](#_Toc82002307)

[1.3.4. Glycine based barbiturate tracer [^99m^Tc]MEA39 32](#_Toc82002308)

[1.3.5. Lysine based barbiturate tracer [^99m^Tc]MEA61 35](#_Toc82002309)

[1.3.6. HYNIC based barbiturate tracer [^99m^Tc]MEA223 38](#_Toc82002310)

[1.4. Western blot 41](#_Toc82002304)

[2. Bibliography 4](#_Toc82002311)2

# Experimental procedures

## General considerations

Materials and Methods.

All *chemicals*, *reagents*, and *solvents* for the syntheses of the compounds were analytical grade, purchased from commercial sources and used without further purification unless otherwise specified. Solvents were purified and dried by literature procedures if necessary. Only solvents of pharmaceutical purity from ABX and Milli-Q^®^-water or water for injection from B. Braun were used for radiosynthesis.

*Chemical yields* were calculated relative to the minor reactant.

The *melting points* (mp) were uncorrected and were determined in capillary tubes on a Stuart Scientific SMP3 capillary melting point apparatus.

*Preparative column chromatography* was performed using Merck silica gel 60 (0.040-0.063 mm). The volume fractions and the corresponding solvents used for purification can be found at the experiments. In some cases, automated column chromatography utilizing a Reveleris X2 from Grace (now Büchi) was performed. The relevant parameters may be found in form of tables at the experiments, the general methods may be found below:

- method I: A 4 g Reveleris^®^C18 reversed-phase flash cartridge was calibrated using the starting volume fraction of solvents for 2.4 min. The method used a linear gradient from 0% to 12% acetonitrile in water over 9 min, followed by a gradient of 12% to 100% acetonitrile in water over 4 min followed by pure acetonitrile for 2 min. The flow rate was set to 18 mL/min.
- method II: A 12 g Reveleris^®^C18 reversed-phase flash cartridge was calibrated using the starting volume fraction of solvents for 4.8 min. The method used a linear gradient from 0% to 10% acetonitrile in water over 7 min, followed by a gradient of 10% to 100% acetonitrile in water over 5 min followed by pure acetonitrile for 2 min. A gradient of 100% to 0% acetonitrile was applied over 2 min and the column was flushed with water for 2 min. The flow rate was set to 30 mL/min.
- method III: A 4 g Reveleris^®^C18 reversed-phase flash cartridge was calibrated using the starting volume fraction of solvents for 2.4 min. The method used a linear gradient from 0% to 10% acetonitrile in water over 4 min, followed by a gradient of 10% to 100% acetonitrile in water over 3 min followed by pure acetonitrile for 2 min. A gradient of 100% to 0% acetonitrile was applied over 2 min and the column was flushed with water for 2 min. The flow rate was set to 18 mL/min.

*Thin layer chromatography* (TLC) was carried out on silica gel-coated polyester backed TLC plates (Polygram, SIL G/UV_254_, Macherey-Nagel) using solvent mixtures of cyclohexane (CH), ethyl acetate (EA), dichloromethane (CH_2_Cl_2_) and methanol (MeOH). Detection of compounds was accomplished using UV-light (wave length = 254 nm or 366 nm) or using different reagent solutions followed by heating to 250°C:

- Cerammoniummolybdate-reagent (1.0 g cer-(IV)-sulfate, 2.5 g molybdatophosphoric acid, 6 mL concentrated sulfuric acid solved in 94 mL distilled water)

- Permanganate-solution (3 g potassium permanganate, 0.25 g sodium hydroxide, 20 g sodium carbonate solved in 300 mL distilled water)

The *NMR spectra* were recorded on Bruker AV300 MHz, Bruker AV400 MHz and Agilent DD2 600 spectrometers. Chemical shifts are reported on the *δ* scale relative to tetramethylsilane (0 ppm) or using solvent signals for calibration. ^1^H NMR spectra were referenced relative to the used deuterated solvents or TMS as the internal standard, ^13^C NMR spectra were referenced to the used deuterated solvents and are decoupled. Analysis and processing of NMR-spectra was performed using MestReNova (version 10.0) from Mestrelab Research S.L.

*Mass spectra* were recorded on the following devices using electrospray ionization (ESI):

- MicroTof (Bruker Daltonics)

- Orbitrap LTQ XL (Thermo Scientific)

*Exact masses* were determined using one of the following devices and methods:

- QUATTRO LCZ (Waters-Micromass), nanospray, ESI

- MicroTof (Bruker Daltonics), loop inlet, ESI. Calibration was performed directly prior to measurements using sodium formiate clusters.

- Orbitrap LTQ XL (Thermo Scientific), loop inlet, ESI.

- AutoFlex Speed (Bruker Daltonics), Multiple Target Plate (MTP) or Prespotted Achor Chip (PAC) and matrix assisted laser desorption/ionization (MALDI).

*Crystal structure analysis* was done using a Bruker Kappa CCD APEXII diffractometer with a standard copper source. The following software was used for evaluation and processing of generated data: COLLECT Bruker AXS, Denzo-SMN, SHELXS-97, SHELXL-97, XP from Bruker AXS.

*Separation*, *purification* and determination of *(radio)chemical purity* of the compounds were performed by using the preparative, semipreparative and analytical reversed-phase HPLC systems A, B and C.

- Preparative HPLC A: Two Wellchrome preparative K-1800 pumps, a Smartline UV detector 2500 (Herbert Knauer GmbH) and a Eurospher-100-C18 column (20 mm × 250 mm). The recorded data were processed by the ChromGate HPLC software (Herbert Knauer GmbH). The HPLC method started with 10% CH_3_CN in water (0.1% TFA) over 7 min, followed by a linear gradient from 10% to 80% CH_3_CN in water (0.1% TFA) from 7-18 min, then isocratic for 20 min followed by a linear gradient from 80% to 10% CH_3_CN in water (0.1% TFA) over 2 min. The flow rate was 7 mL·min^−1^.
- Semipreparative HPLC B: A Wellchrome K-500 pump and a Wellchrome K-501 pump, a K-2000 UV detector (Herbert Knauer GmbH), a NaI(TI) Scintibloc 51 SP51 γ-detector (Crismatec), and a ACE 5 AQ column (10 mm × 250 mm). The recorded data was processed by the GINA Star software (Raytest Isotopenmessgeräte GmbH). The HPLC method B1 started with a linear gradient from 10% to 60% CH_3_CN in water (0.1% TFA) over 25 min, isocratic for 5 min followed by a linear gradient from 60% to 10% CH_3_CN in water (0.1% TFA) over 5 min, then isocratic for 5 min. The flow rate was 7.0 mL·min^−1^. HPLC method B2 started with a linear gradient from 20% to 65% CH_3_CN in water (0.1% TFA) over 38 min, isocratic for 7 min followed by a linear gradient from 65% to 10% CH_3_CN in water (0.1% TFA) over 5 min. The flow rate was 5.5 mL·min^−1^.
- Analytical HPLC C: Two Smartline 1000 pumps and a Smartline UV detector 2500 (Herbert Knauer GmbH), a GabiStar γ-detector (Raytest Isotopenmessgeräte GmbH) and a Nucleosil 100-5 C–18 column (4 mm × 250 mm). The recorded data was processed by the GINA Star software (Raytest Isotopenmessgeräte GmbH). The HPLC method started with a linear gradient from 10% to 90% CH_3_CN in water (0.1% TFA) over 9 min, followed by a linear gradient from 90% to 10% CH_3_CN in water (0.1% TFA) over 6 min. The flow rate was 1 mL·min^−1^.

*Lyophilisation* of compounds which have been purified by reversed-phase HPLC or RP-Reveleris® chromatography was performed using an Alpha 2-4 LD plus-freeze-dryer from Christ.

Methyl *N*^2^-(*tert*-butoxycarbonyl)-*N*^2^-(prop-2-yn-1-yl)-*N*^6^-(2,2,2-trifluoroacetyl)-L-lysinate (**8**)[1], 5-(4-(2-(2-(2-(2-Azidoethoxy)ethoxy)ethoxy)ethyl)piperazin-1-yl)-5-(4-phenoxyphenyl)-pyrimidine-2,4,6-trione (**11**)[2] and [NEt_3_]_2_[ReBr_3_(CO)_3_] [3] were synthesized following literature procedures.

## Synthetic Organic Chemistry / Spectra of new compounds

### Synthesis of spacer units and general side chain building blocks

#### Ethyl methanesulfonic acid-2-{2-[2-(2‑methanesulfonyloxyethoxy)ethoxy]ethoxy}carboxylate (1)

Triethylamine (79.7 mL, 58.2 g, 575 mmol, 2.3 eq.) was slowly added to a solution of tetraethylene glycole (48.6 g, 250 mmol, 1 eq.) in dichloromethane (500 mL) at 0 °C. A solution of methanesulfonic acid chloride (44.5 mL, 65.9 g, 575 mmol, 2.3 eq.) in dichloromethane (25 mL) was added dropwise and the reaction was stirred at room temperature overnight. The precipitate was filtered off, the filtrate was washed with brine (250 mL) and dried over magnesium sulfate. The solvent was removed under reduced pressure and the residue was purified by column chromatography using silica (solvent: ethyl acetate). The product was isolated as a colorless oil. **Yield:** 72.9 g (208 mmol, 83%). **^1^H-NMR** (300 MHz, CDCl_3_): δ (ppm) 3.05 (s, 6H), 3.59-3.65 (m, 8H), 3.73‑3.76 (m, 4H), 4.34-4.37 (m, 4H). **^13^C-NMR** (75 MHz, CDCl_3_): δ (ppm) 37.7, 69.1, 69.4, 70.5, 70.7. **MS-ESI(+):** *m/z* = 373.0604 [M+Na]^+^. All analytical data are in agreement with the previously reported data.[4]

#### 2-{2-[2-(2-Azidoethoxy)ethoxy]ethoxy}ethyl methanesulfonate (2)

Ethyl methanesulfonic acid-2-{2-[2-(2‑methanesulfonyloxyethoxy)ethoxy]ethoxy} carboxylate (**1**) (64.0 g, 183 mmol, 1 eq.) was slowly added to a suspension of sodium azide (11.9 g, 183 mmol, 1 eq.) in dimethylformamide (540 mL) and stirred at room temperature for 78 h. Water (360 mL) was added and the mixture was extracted with ethyl acetate (3 × 500 mL). The combined organic layers were washed with water and brine (600 mL each), dried over magnesium sulfate and the solvent was removed under reduced pressure. The residue was purified by column chromatography using silica (solvent: cyclohexane:ethyl acetate 2:1 gradient to 1:3). The product was isolated as a colorless oil. **Yield:** 24.2 g (81.0 mmol, 44%). **^1^H-NMR** (300 MHz, CDCl_3_): δ (ppm) 3.04 (s, 3H), 3.35-3.38 (m, 2H), 3.61‑3.65 (m, 10H), 3.71-3.74 (m, 2H), 4.32-4.35 (m, 2H). **^13^C-NMR** (75 MHz, CDCl_3_): δ (ppm) 37.8, 50.6, 69.0, 69.4, 70.0, 70.5, 70.6, 70.7. **MS-ESI(+):** *m/z* = 320.0889 [M+Na]^+^. All analytical data are in agreement with the previously reported data.[4]

#### 1-(2-{2-[2-(2-Azidoethoxy)ethoxy]ethoxy}ethyl)piperazine (3).

To a solution of 2-{2-[2-(2-azidoethoxy)ethoxy]ethoxy}ethyl methanesulfonate (**2**) (3.00 g, 14 mmol, 1 eq.) in acetonitrile (90 mL) piperazine (2.65 g, 31 mmol, 2.2 eq.) and triethylamine (4.27 mL, 3.12 g, 31 mmol, 2.2 eq.) were added. The reaction mixture was stirred at room temperature for 6 h followed by heating to reflux for 12 h. After removing of the solvent under reduced pressure, the resulting oil was dissolved in chloroform (15 mL) and washed with saturated sodium bicarbonate solution (20 mL). The aqueous layer was extracted with chloroform (3 × 15 mL), the combined organic layers were dried over magnesium sulfate and the solvent was removed under reduced pressure. The residue was purified using a silica gel column (EtOAc:MeOH 9:1 gradient to 4:1) to give a yellow, sticky oil. **Yield:** 1.73 g (6.0 mmol, 43%). **^1^H-NMR** (MeOD, 300 MHz): δ (ppm) 2.59-2.78 (m, 6H), 3.01‑3.10 (m, 4H), 3.37 (t, ^3^*J* = 4.7 Hz, 2H), 3.59-3.72 (m, 12H). **^13^C-NMR** (MeOD, 75 MHz): δ (ppm) 39.5, 45.3, 51.8, 52.9, 58.7, 69.6, 71.1, 71.4, 71.6, 71.6. **MS-ESI(+):** *m/z* = 310.1854 [M + Na]^+^, 288.2034 [M + H]^+^.

#### (*S*)-Methyl-2-[(*tert*-butoxycarbonyl)amino]-6-(2,2,2-trifluoroacetamido)hexanoate (5)

Trifluoroacetic acid anhydride (4.49 mL, 6.68 g, 31.8 mmol, 3 eq.) was slowly added (2 h) to a cooled (0 °C) solution of Boc-Lys-OMe **^.^** AcOH (68%, 5.00 g, 10.6 mmol, 1 eq.) and diisopropylethyl amine (7.21 mL, 5.48 g, 42.4 mmol, 4 eq.) in tetrahydrofuran (80 mL). After addition the solution was stirred at room temperature for 12 h. The volume of the solvent was reduced to 40 mL under reduced pressure and water (75 mL) was added. The mixture was extracted with ethyl acetate (3 × 100 mL). The combined organic layers were washed with water and brine (100 mL each), dried over magnesium sulfate and the solvent was removed under reduced pressure. The residue was purified by column chromatography using silica (solvents: cyclohexane:ethyl acetate 4:1). The product was isolated as a colorless oil. **Yield:** 2.90 g (8.14 mmol, 78%). **^1^H-NMR** (400 MHz, CDCl_3_): δ = 1.43 (s, 9H), 1.30-1.44 (m, 2H), 1.58-1.70 (m, 3H), 1.76-1.86 (m, 1H), 3.36 (q, ^3^*J* = 6.4 Hz, 2H), 3.74 (s, 3H), 4.23-4.36 (m, 1H), 5.10 (bd, *J* = 7.7 Hz, 1H), 6.66 (s, 1H). **^13^C-NMR** (100 MHz CDCl_3_): δ = 22.5, 28.3, 28.4, 32.7, 39.8, 52.5, 53.0, 80.3, 115.5 (q, *J*_C-F_ = 285 Hz), 155.7, 157.6 (q, *J*_C-F_ = 35 Hz), 173.2. **MS-ESI(+):** *m/z* = 379.1450 [M+Na]^+^, 735.3003 [2M+Na]^+^. All analytical data are in agreement with the previously reported data.[5]

#### (*S*)-Methyl-6-(2,2,2-trifluoroacetamido)hexanoate TFA salt (6)

Trifluoroacetic acid (81.0 mL, 119.9 g, 1.05 mol, 161 eq.) was added to a solution of (*S*)-methyl-2-[(*tert*-butoxycarbonyl)amino]-6-(2,2,2-trifluoroacetamido)hexanoate (**5**) (2.33 g, 6.53 mmol, 1 eq.) in dichloromethane (325 mL) and stirred at room temperature for 18 h. Solvents were removed under reduced pressure and the product (yellow oil) was used without further purification. **Yield:** 1.54 g (4.16 mmol, 64%). **^1^H-NMR** (300 MHz, CD_3_OD): δ = 1.28-1.48 (m, 2H), 1.54 (quintt, ^3^*J* = 7.2 Hz, 2H), 1.75-1.96 (m, 2 H), 3.22-3.26 (m, 2H, overlay with solvent signal), 3.77 (s, 3 H), 3.97 (t, ^3^*J* = 6.4 Hz, 1H). **^13^C-NMR** (75 MHz, CD_3_OD): δ = 23.1, 29.3, 31.0, 40.1, 53.6, 53.8, 116.7 (q, *J*_C-F_ = 285 Hz), 159.3 (q, *J*_C-F_ = 35 Hz), 170.9. **MS-ESI(+):** *m/z* = 257.1152 [M+H]^+^, 279.0926 [M+Na]^+^. All analytical data are in agreement with the previously reported data.[5]

#### (*S*)-Methyl-2-(prop-2-yn-1-yl)-6-(2,2,2-trifluoroacetamido)hexanoate (7)

Diisopropylethylamine (5.56 mL, 4.39 g, 34.0 mmol, 3.6 eq.) was added to a solution of (*S*)-methyl-6-(2,2,2-trifluoroacetamido)hexanoate TFA salt (**6**) (3.48 g, 9.4 mmol, 1 eq.) in tetrahydrofuran (200 mL). After addition, propargyl bromide (80% in toluen, 3.02 mL, 4.04 g, 27.2 mmol, 2.9 eq.) was added and the resulting mixture was stirred at room temperature for 2 d. The solvents were removed under reduced pressure and the residue was purified by column chromatography using silica (solvents: cyclohexane:ethyl acetate 1:1). The product was isolated as a slightly yellow oil. **Yield:** 1.40 g (4.78 mmol, 51%). **^1^H-NMR** (300 MHz, CDCl_3_): δ = 1.37-1.48 (m, 2 H), 1.56-1.72 (m, 4 H), 2.03 (br s, 1 H), 2.22 (t, ^3^*J* = 2.5 Hz, 1H), 3.32-3.49 (m, 5 H), 3.73 (s, 3H), 6.72 (br s, 1 H). **^13^C-NMR** (75 MHz, CDCl_3_): δ = 22.7, 28.4, 32.3, 37.0, 39.7, 52.1, 59.7, 72.1, 81.1, 115.9 (q, *J*_C-F_ = 285 Hz), 157.6 (q, *J*_C-F_ = 35 Hz), 175.1. **MS-ESI(+):** *m/z* = 295.1264 [M+H]^+^, 317.1079 [M+Na]^+^. All analytical data are in agreement with the previously reported data.[5]

#### (*S*)-Methyl-2-(*tert*-butoxycarbonyl)-2-(prop-2-yn-1-yl)-6-(2,2,2-trifluoroacetamido)hexanoate (8)

(*S*)-Methyl-2-(prop-2-yn-1-yl)-6-(2,2,2-trifluoroacetamido)hexanoate (**7**) (1.19 g, 4.03 mmol, 1 eq.) was dissolved in tetrahydrofuran (20 mL) and diisopropylamine (1.75 mL, 1.30 g, 10.1 mmol, 2.5 eq.) and di-*tert*-butyldicarbonate (1.32 g, 6.04 mmol, 1.5 eq.) were added. The solution was stirred at 55°C for 4 d and concentrated under reduced pressure. Purification of the residue by column chromatography on silica gel with cyclohexane:acetone (9:1) gave the product as a yellow oil. **Yield:** 1.48 mg (3.75 mmol, 93%). **^1^H-NMR** (400 MHz, CDCl_3_): δ = 1.23-1.40 (singlet overlayed by m, 11H), 1.48-1.70 (m, 3H), 1.96 (s, 1H), 3.26-3.50 (m, 3H), 3.51-3.54 (m, 1H), 3.61 (s, 3H), 4.03 (q, *J* = 7.2 Hz, 1H), 7.33-7.26 (m, 1H). **^13^C-NMR** (100 MHz, CDCl_3_): δ = 22.8, 27.1, 28.2, 29.0, 34.1, 39.6, 51.3, 60.4, 72.8, 79.2, 81.5, 115.9 (q, *J*_C-F_ = 288 Hz), 151.4, 157.3 (q, *J*_C-F_ = 35 Hz), 172.7. **MS-ESI(+):** *m/z* = 417.1611 [M+Na]^+^, 811.3324 [2M+Na]^+^. All analytical data are generally in agreement with the previously reported data.[5]

### Synthesis of central pyrimidine-2,4,6-trione building blocks

#### Diisopropyl-2-(4-phenoxyphenyl)malonate (9)

A mixture of palladium acetate (135 mg, 0.6 mmol, 8 mol%), copper difluoride (60 mg, 0.6 mmol, 8 mol%), tri-*tert*-butyl phosphine (90% grade, 220 μL, 0.8 mmol, 10 mol%), sodium-*tert*-butoxide (6.98 g, 73 mmol, 1.2 eq.) and tetrahydrofuran (30 mL) was prepared. 4-Bromophenyl phenylether (14.9 g, 10.5 mL, 60 mmol, 1 eq.) and diisopropyl malonate (14.3 g, 14.5 mL, 76 mmol, 1.26 eq.) were added consecutively. The solution was heated to 67 °C for 16 h. The resulting suspension was diluted with cyclohexane (30 mL), and 3 M hydrochloric acid (7.5 mL, 22.5 mmol) was added. The mixture was filtered through Celite, and the filter pad was washed with cyclohexane:tetrahydrofuran (1:1, 100 mL). After removing the solvent, the residue was dissolved in diethylether (50 mL), washed with 3 M hydrochloric acid (3 × 25 mL), 1 M sodium bicarbonate solution (2 × 25 mL) and brine (25 mL), dried over magnesium sulfate and concentrated under reduced pressure yielding a yellow oil. The product was used in the next step without further purification. **Yield:** 19.7 g (55.0 mmol, 92%). **^1^H-NMR** (300 MHz, CDCl_3_): δ = 1.23-1.28 (m, 12H), 4.52 (s, 1H), 5.07 (hept, *J* = 6.3 Hz, 2H), 6.95-7.05 (m, 4H), 7.08-7.14 (m, 1H), 7.30-7.38 (m, 4H). **^13^C-NMR** (75 MHz, CDCl_3_): δ = 21.7, 21.7, 21.8, 57.8, 69.5, 118.6, 119.4, 123.6, 129.8, 129.9, 130.8, 156.9, 157.4, 167.9. **MS-ESI(+):** *m/z* = 379.1523 [M+Na]^+^, 735.3130 [2M+Na]^+^. All analytical data are in agreement with the previously reported data.[6]

#### 5-(4-Phenoxyphenyl)pyrimidine-2,4,6-trione (10)

Urea (6.60 g, 110 mmol, 2 eq.) and isopropanol (130 mL) were added to diisopropyl-2-(4-phenoxyphenyl)malonate (**9**) (19.7 g, 55.0 mmol, 1 eq.). The mixture was heated to 85 °C and a 1M solution of potassium-*tert*-butoxide in tetrahydrofuran (165 mL, 165 mmol, 3 eq.) was added slowly over 5 h. The mixture was heated to reflux for additional 5 h and was stirred overnight. After cooling to room temperature, concentrated hydrochloric acid (15 mL) and water (35 mL) were slowly added and the mixture was stirred for 10 min. The solution was concentrated under reduced pressure to remove the organic solvents. The resulting aqueous suspension was diluted with water (100 mL), and the precipitate was filtered off. The off-white solid was dissolved in ethyl acetate (70 mL), heated to reflux for a few minutes, and after cooling to ambient temperature cyclohexane (35 mL) was slowly added. The precipitate was filtered off, washed with cyclohexane:ethyl acetate (1:1, 100 mL), and dried to give an off-white solid. **Yield:** 9.62 g (33.0 mmol, 60%). **^1^H-NMR** (300 MHz, DMSO-d6): δ = 4.85 (s, 1H), 6.95-6.98 (m, 2H), 7.01-7.04 (m, 2H), 7.13‑7.18 (m, 1H), 7.26-7.28 (m, 2H), 7.37‑7.43 (m, 2H), 10.76 (s, 1H), 11.39 (s, 1H). **^13^C-NMR** (75 MHz, DMSO-d6): δ = 54.4, 118.4, 119.0, 123.8, 129.2, 130.2, 131.1, 151.0, 156.3, 156.4, 169.3. **MS-ESI(+):** *m/z* = 297.08701 [M+H]^+^. All analytical data are in agreement with the previously reported data.[6]

#### 5-[4-(2-{2-[2-(2-Azidoethoxy)ethoxy]ethoxy}ethyl)piperazin-1-yl]-5-(4-phenoxyphenyl)-pyrimidine-2,4,6-trione (11).

A solution of 5-(4-phenoxyphenyl)-pyrimidine-2,4,6-trione (**10**) (1.78 g, 6.02 mmol, 1 eq.) in dimethyl formamide (10 mL) was cooled to 0 °C, and a solution of *N*-bromosuccinimide (1.07 g, 6.02 mmol, 1 eq.) in dimethyl formamide (10 mL) was added over 20 min while maintaining the temperature of the reaction mixture below 10 °C. After stirring for 20 min, 1-(2-{2-[2-(2-azidoethoxy)ethoxy]ethoxy}ethyl)piperazine (**3**) (1.73 g, 6.02 mmol, 1 eq.) in dimethyl formamide (10 mL) and potassium carbonate (2.08 g, 15.05 mmol, 2.5 eq.) were added, and the mixture was stirred for 1 h at 0 °C followed by stirring for 20 h at room temperature. After removing of the solvent, the crude product was suspended in acetone (25 mL). The precipitate was filtered off, dried and purified by column chromatography on silica gel (dichloromethane:methanol 9:1) to give a pale yellow solid. **Yield:** 2.56 g (4.4 mmol, 73%). **^1^H-NMR** (300 MHz, DMSO-d_6_): δ (ppm) 2.45-2.58 (m, 10H), 3.37 (t, ^3^*J* = 5.2 Hz, 2H), 3.47-3.60 (m, 12H), 7.00-7.03 (m, 2H), 7.04-7.07 (m, 2H), 7.15‑7.20 (m, 1H), 7.38-7.43 (m, 4H), 11.61 (s, 2H). **^13^C-NMR** (75 MHz, DMSO-d_6_): δ (ppm) 47.3, 50.0, 53.7, 57.1, 68.1, 69.3, 69.6, 69.7, 69.8, 73.9, 118.1, 118.2, 118.5, 119.3, 124.1, 127.7, 129.6, 129.7, 130.2, 149.4, 155.8, 157.4, 170.0. **MS-ESI(+):** *m/z* = 604.2477 (M + Na)^+^, 582.2658 (M + H)^+^. All analytical data are in agreement with the previously reported data.[4]

##### Crystal structure of 5-[4-(2-{2-[2-(2-Azidoethoxy)ethoxy]ethoxy}ethyl)piperazin-1-yl]-5-(4-phenoxyphenyl)pyrimidine-2,4,6-trione (11)


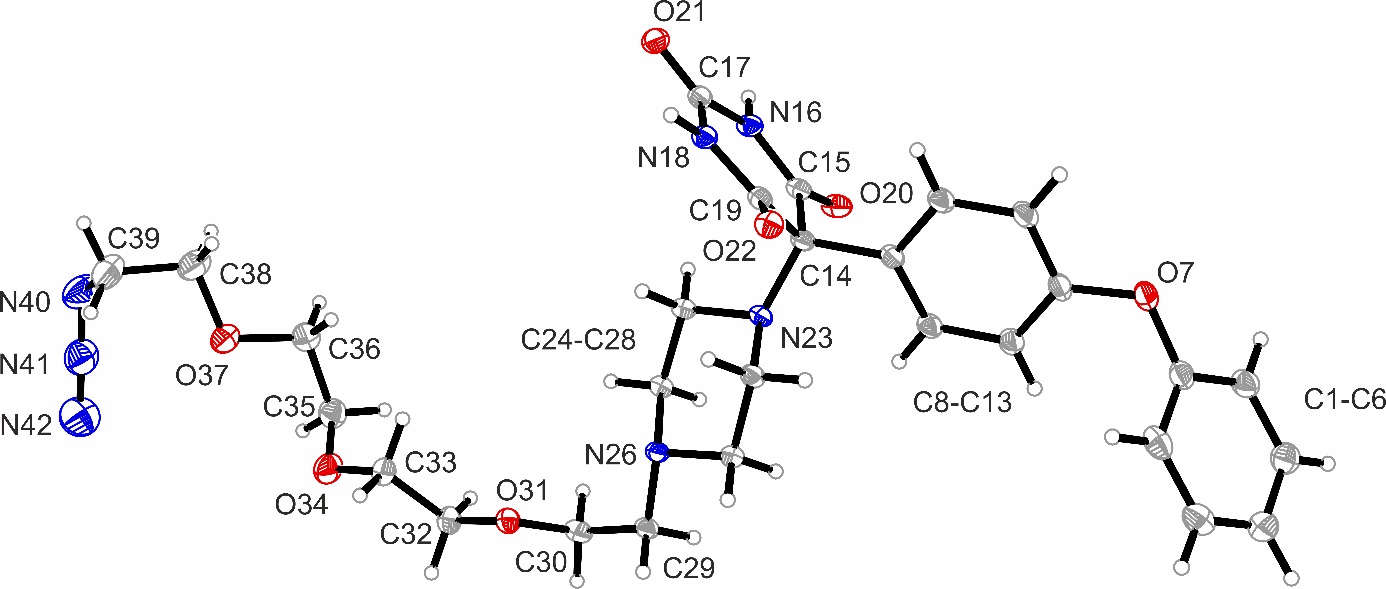


Figure 1: Crystal structure of compound 11, Thermal ellipsoids are shown at 15% probability.

CCDC 2090342 for compound **11** is contained in the supplementary crystallographic data for this manuscript. This data can be obtained free of charge from The Cambridge Crystallographic Data Centre *via* www.ccdc.cam.ac.uk/data_request/cif.

Datablock: haf6800

Bond precision: C-C = 0.0047 A; Wavelength=1.54178

Cell parameters: a=15.036(2) b=9.7779(8) c=21.0670(15)

alpha=90 beta=109.223(6) gamma=90

Temperature: 223 K

Table 1: Most important parameters of refinement

|  | Calculated | Reported |
| --- | --- | --- |
| Volume | 2924.6 (5) | 2924.6 (5) |
| Space group | P 21/c | P1 21/c 1 |
| Hall group | -P 2ybc | -P 2ybc |
| Moiety formula | C_28_H_35_N_7_O_7_ | ? |
| Sum formula | C_28_H_35_N_7_O_7_ | C_28_H_35_N_7_O_7_ |
| M_r_ | 581.63 | 581.63 |
| Dx, g/cm³ | 1.321 | 1.321 |
| Z | 4 | 4 |
| M_u_ (1/mm) | 0.804 | 0.804 |
| F000 | 1232.0 | 1232.0 |
| F000´ | 1236.01 |  |
| h,k,l_max_ | 17,11,25 | 17,11,24 |
| N_ref_ | 5222 | 4990 |
| T_min_, T_max_ | 0.891, 0.961 | 0.820, 0.960 |
| T_min_´ | 0.818 |  |

Correction method= # Reported T Limits: T_min_ = 0.820 T_max_ = 0.960

AbsCorr = MULTI-SCAN

Data completeness= 0.956 Theta(max)= 67.020

R(reflections)= 0.0580(3530) wR2(reflections)= 0.1551(4990)

S = 1.027 N_par_= 406

**X-Ray diffraction:** For compound **11** data sets were collected with a Nonius Kappa CCD diffractometer. Programs used: data collection, COLLECT[7]; data reduction Denzo-SMN[8]; absorption correction, Denzo[9]; structure solution *SHELXT-2015*[10]; structure refinement *SHELXL-2015*[11] and graphics, *XP*[12]. *R*-values are given for observed reflections, and *w*R^2^ values are given for all reflections.

*Exceptions and special features*: For compound **11** one ethoxy unit was found disordered over two positions in the asymmetric unit. Several restraints (SADI, SAME, ISOR and SIMU) were used in order to improve refinement stability. The hydrogens at N16 and N18 atoms were refined freely, but with N-H distance restraint (DFIX).

**X-ray crystal structure analysis of 11 (haf6800):** A colorless plate-like specimen of C_28_H_35_N_7_O_7_, approximate dimensions 0.050 mm x 0.120 mm x 0.250 mm, was used for the X-ray crystallographic analysis. The X-ray intensity data were measured on a Nonius KappaCCD APEXII system equipped with a Cu fine-focus sealed tube fine-focus sealed tube ('CuKα', λ = 1.54178 Å) and a graphite monochromator. The integration of the data using a monoclinic unit cell yielded a total of 4990 reflections to a maximum θ angle of 67.02° (0.84 Å resolution), of which 4990 were independent (average redundancy 1.000, completeness = 95.6%, R_sig_ = 3.69%) and 3530 (70.74%) were greater than 2σ(F^2^). The final cell constants of a = 15.036(2) Å, b = 9.7779(8) Å, c = 21.0670(15) Å, β = 109.223(6)°, volume = 2924.6(5) Å^3^, are based upon the refinement of the XYZ-centroids of reflections above 20 σ(I). Data were corrected for absorption effects using the multi-scan method (SADABS). The calculated minimum and maximum transmission coefficients (based on crystal size) are 0.8240 and 0.9610. The structure was solved and refined using the Bruker SHELXTL Software Package, using the space group *P*2_1_/*c*, with Z = 4 for the formula unit, C_28_H_35_N_7_O_7_. The final anisotropic full-matrix least-squares refinement on F^2^ with 406 variables converged at R1 = 5.80%, for the observed data and wR2 = 15.51% for all data. The goodness-of-fit was 1.027. The largest peak in the final difference electron density synthesis was 0.219 e^-^/Å^3^ and the largest hole was -0.263 e^-^/Å^3^ with an RMS deviation of 0.043 e^-^/Å^3^. On the basis of the final model, the calculated density was 1.321 g/cm^3^ and F(000), 1232 e^-^. The hydrogens at N16 and N18 atoms were refined freely, but with N-H distance restraint (DFIX). CCDC Nr.: 2090342.

#### 5-[4-(2-{2-[2-(2-Aminoethoxy)ethoxy]ethoxy}ethyl)piperazine-1-yl]-5-(4-phenoxyphenyl)-pyrimidine-2,4,6-trione (12)

Triphenylphosphane (1.38 g, 5.28 mmol, 2.5 eq.) was added to a solution of 5-[4-(2-{2-[2-(2-azidoethoxy)ethoxy]ethoxy}ethyl)piperazine-1-yl]-5-(4-phenoxyphenyl)pyrimidine-2,4,6-trione (**11**) (1.23 g, 2.11 mmol, 1 eq.) in tetrahydrofuran:water (3:1, 60 mL). The solution was stirred for 2 d at room temperature, the solvents were removed under reduced pressure and the residue was purified by column chromatography using deactivated silica (pretreatment with 2.5% triethylamine in ethyl acetate, solvent gradient moving from ethyl acetate to ethyl acetate:methanol 4:1). The product was isolated as a colorless solid. **Yield:** 678 mg (1.22 mmol, 58%). **^1^H-NMR** (400 MHz, CD_3_OD): δ = 2.54-2.57 (m, 6H), 2.73-2.74 (m, 4H), 2.93-2.96 (m, 2H), 3.54-3.59 (m, 12H), 6.88‑6.90 (m, 2H), 6.95‑6.97 (m, 2H), 7.08‑7.11 (m, 1H), 7.30-7.34 (m, 2H), 7.45‑7.47 (m, 2H). **^13^C-NMR** (100 MHz, CD_3_OD): δ = 41.1, 47.0, 54.9, 58.7, 68.9, 70.8, 71.2, 71.4, 71.5, 75.5, 119.1, 120.4, 125.9, 131.0, 131.1, 132.0, 156.8, 157.9, 159.4, 176.1. **MS-ESI(+):** *m/z* = 556.2767 [M+H]^+^, 578.2582 [M+Na]^+^. All analytical data are in agreement with the previously reported data.[4]

### General procedure A: CuAAC of alkines with barbiturate azide (11).

Two solutions of copper(II)sulfate pentahydrate (0.3 eq.) and sodium ascorbate (0.4 eq.) in water (each 0.75 mL/mmol (regarding the 1 eq. compound)) were mixed in a reaction vessel. A solution of 5-[4-(2-{2-[2-(2-azidoethoxy)ethoxy]ethoxy}ethyl)piperazine-1-yl]-5-(4-phenoxyphenyl)pyrimidine-2,4,6-trione (**11**) (1 eq.) in dimethyl formamide (10 mL/mmol) and a solution of the corresponding propargyl amino acid derivative (1-1.1 eq.) in dimethyl formamide (2.5 mL/mmol) were added. The reaction mixture was stirred at room temperature for 12 h. After removing the solvents the crude product was chromatographed on a silica gel column or by HPLC system A or used directly.

### General procedure B: Removal of Boc protecting groups.

A solution of the Boc protected barbiturates (1 eq.) in dichloromethane (50 mL/mmol) was cooled to 0 °C. Then trifluoroacetic acid (11.5 mL/mmol) was added dropwise. After warming to room temperature the reaction mixture was stirred overnight. The solvents were removed under reduced pressure and the crude product was purified in portions by HPLC system A or used directly.

### General procedure C: Synthesis of rhenium complexes.

The metal chelating triazole (1 eq.) was dissolved in methanol (1 mL/25 µmol) and water (1 mL/25 µmol) was added. The solution was treated with [NEt_4_]_2_[ReBr_3_(CO)_3_] (1 eq.). After heating to 70 °C for 5 h and stirring overnight, the solvents were removed under reduced pressure and the crude product was purified by HPLC system A.

### Synthesis and spectroscopic data of the glycine based barbiturate

#### (*S*)-2-[(*tert*-Butoxycarbonyl)amino]-3-[1-(2-{2-[2-(2-{4-[2,4,6-trioxo-5-(4-phenoxyphenyl)-hexahydropyrimidine-5-yl]piperazine-1-yl}ethoxy)ethoxy]-ethoxy}ethyl)-*1H*-1,2,3-triazol-4-yl]propionic acid (13).

According to general procedure **A** from Boc-*L*-propargylglycine (63 mg, 0.55 mmol, 1.1 eq.). Purification by column chromatography with ethyl acetate:methanol (2:1 to 1:2 + 2.5% TFA) gave a yellow solid. **Yield:** 155 mg (0.2 mmol, 40%). **^1^H-NMR** (CD_3_OD, 400 MHz): δ (ppm) 1.40 (s, 9H), 2.63-2.76 (m, 6H), 2.77-2.80 (m, 4H), 3.03-3.15 (m, 1H), 3.24 (dd, *J*= 14.7 Hz, *J* = 4.8 Hz, 1H), 3.53-3.54 (m, 8H), 3.64 (t, ^3^*J*= 5.4 Hz, 2H), 3.84 (t, ^3^*J*= 5.4 Hz, 2H), 4.24 (d, ^3^*J*= 5.6 Hz, 1H), 4.50 (dd, *J*= 5.1 Hz, *J* = 5.7 Hz, 2H), 6.93‑6.99 (m, 2H), 7.00‑7.05 (m, 2H), 7.14-7.17 (m, 1H), 7.34-7.42 (m, 2H), 7.48 (d, ^3^*J*= 8.5 Hz, 2H), 7.77 (s, 1H). **^13^C-NMR** (CD_3_OD, 100 MHz): δ (ppm) 28.8, 30.3, 48.0, 51.3, 55.0, 56.7, 58.3, 68.9, 70.5, 71.3, 71.5, 71.6, 71.6, 76.1, 80.1, 119.3, 120.7, 124.9, 125.2, 130.5, 130.9, 131.1, 145.4, 151.5, 157.4, 157.6, 160.0, 172.3, 178.2. **MS-ESI(+):** *m/z* = 817.3483 (M + Na)^+^, 795.3660 (M + H)^+^. **MS-ESI(-):** *m/z* = 793.3517 (M - H)^-^.

**
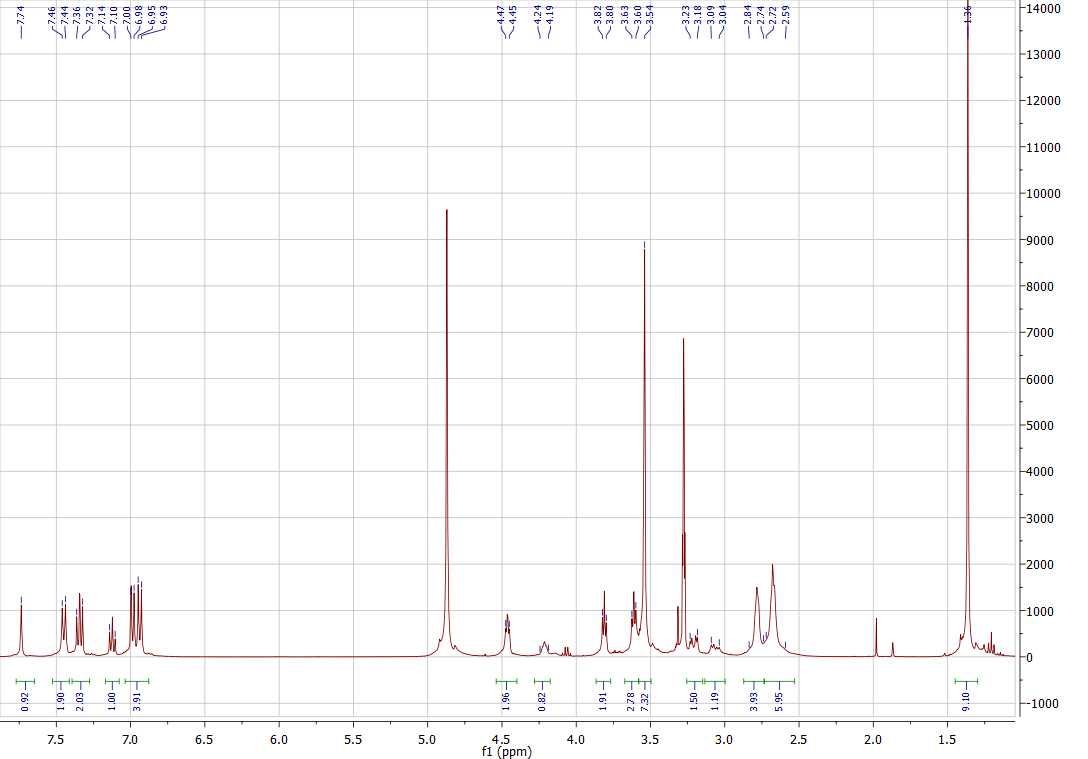
**


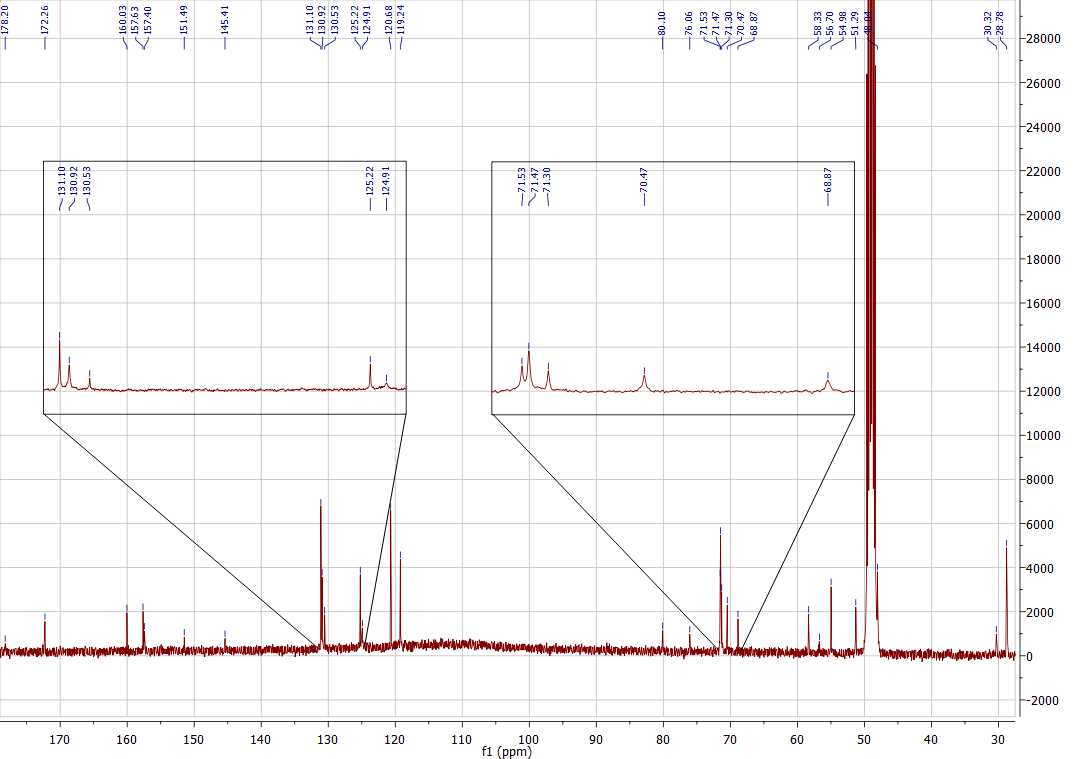


#### (*S*)-2-Amino-3-[1-(2-{2-[2-(2-{4-[2,4,6-trioxo-5-(4-phenoxyphenyl)-hexahydropyrimidine-5-yl]piperazine‑1‑yl}ethoxy)ethoxy]ethoxy}ethyl)-*1H*-1,2,3-triazol-4-yl]propionic acid potassium salt (14).

According to general procedure **B** from (*S*)-2-[(*tert*-butoxycarbonyl)amino]-3-[1-(2-{2-[2-(2-{4-[2,4,6-trioxo-5-(4-phenoxyphenyl)hexahydropyrimidine-5‑yl]piperazine‑1‑yl}ethoxy)ethoxy]-ethoxy}ethyl)-*1H*-1,2,3-triazol-4-yl]propionic acid (**15**) (91 mg, 0.114 mmol, 1 eq.). The crude product was dissolved in tetrahydrofuran and potassium carbonate (200 mg) was added. The mixture was stirred for 5 h, the base was filtered off and the solvent was removed under reduced pressure. Purification of the residue by HPLC system A (no TFA) provided the product as a white solid. **Yield:** 13 mg (0.018 mmol, 16%). **^1^H-NMR** (600 MHz, CD_3_OD): δ (ppm) 2.92-2.94 (m, 2H), 3.13-3.21 (m, 4H), 3.33-3.42 (m, 4H), 3.53-3.54 (m, 2H), 3.59-3.68 (m, 8H), 3.79-3.81 (m, 2H), 3.87 (t, ^3^*J*= 5.4 Hz, 2H), 4.34 (dd, *J* = 5.1 Hz, ^3^*J* = 7.1 Hz, 1H), 4.56 (dd, *J*= 5.1 Hz, *J* = 5.6 Hz, 2H), 7.00‑7.06 (m, 4H), 7.16-7.22 (m, 1H), 7.37-7.40 (m, 2H), 7.48 (d, *J*= 8.7 Hz, 2H), 7.90 (s, 1H). **^13^C-NMR** (150 MHz, CD_3_OD): δ (ppm) 27.2, 46.2, 51.3, 53.7, 54.2, 57.3, 65.2, 70.3, 71.2, 71.3, 71.4, 71.4, 76.2, 119.5, 120.9, 125.4, 125.5, 129.5, 130.6, 131.2, 142.4, 150.6, 157.3, 160.7, 170.8, 171.5. **MS-ESI(+):** *m/z* = 717.2967 (M + Na)^+^, 695.3148 (M + H)^+^.


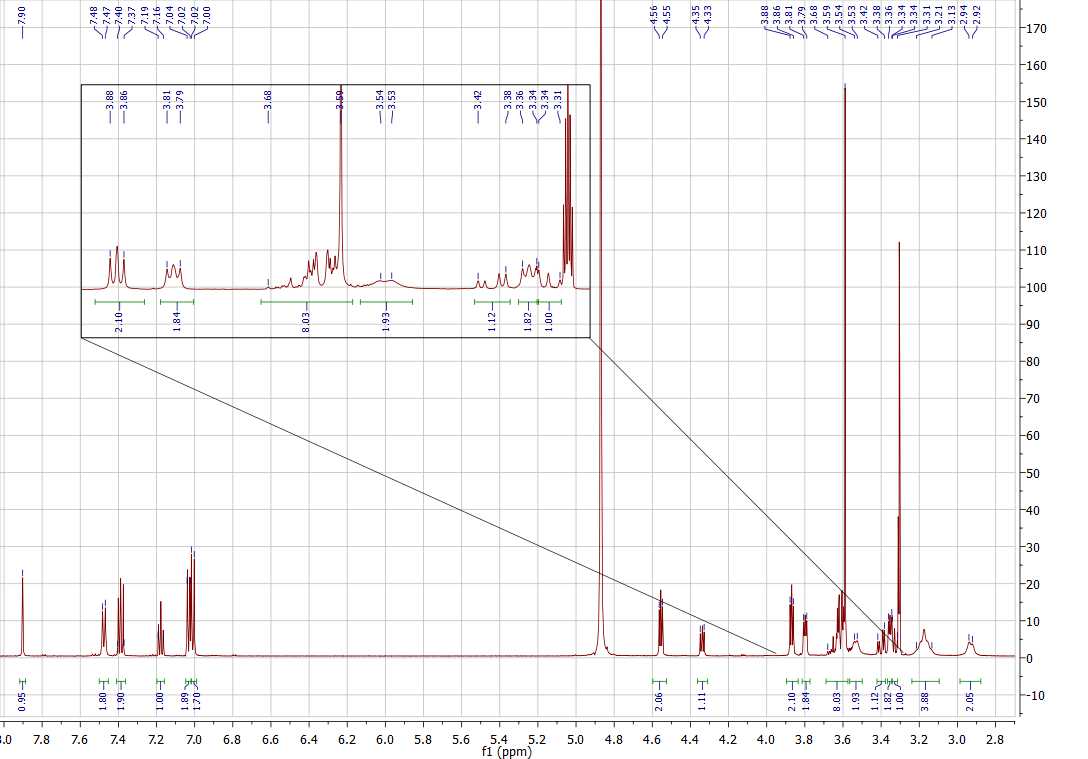


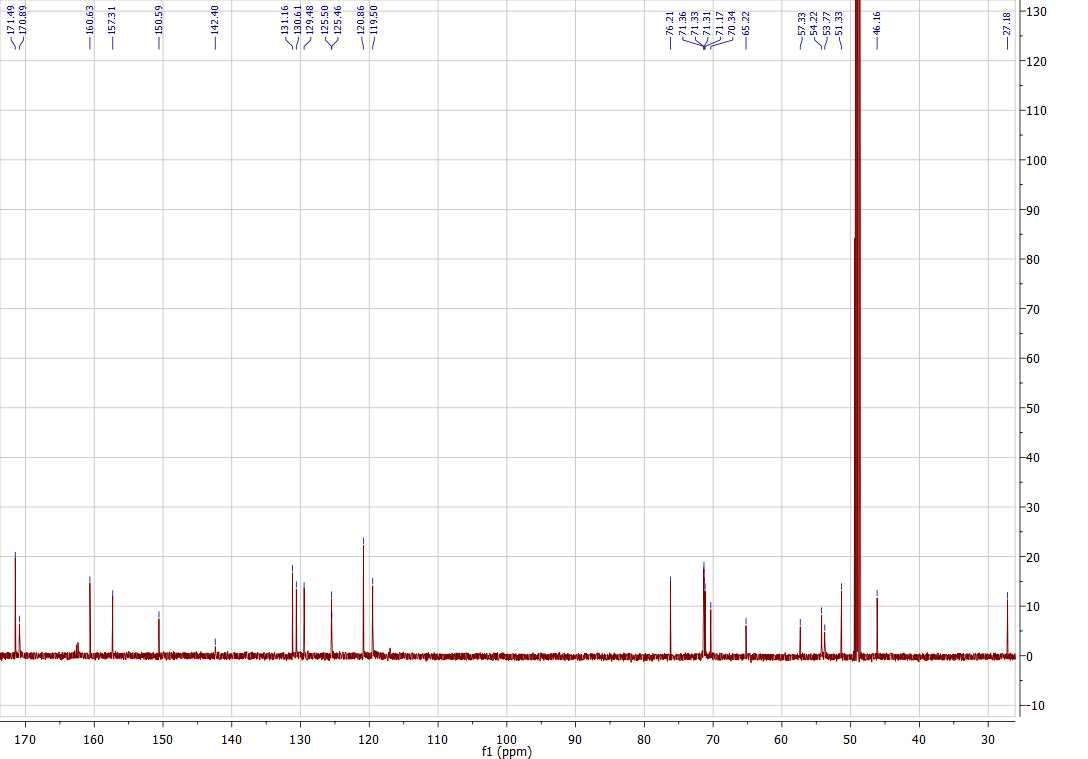


#### (*S*)-2-Amino-3-[1-(2-{2-[2-(2-{4-[2,4,6-trioxo-5-(4-phenoxyphenyl)-hexahydropyrimidine-5-yl]piperazine‑1‑yl}ethoxy)ethoxy]ethoxy}ethyl)-*1H*-1,2,3-triazol-4-yl]propionic acid-carbonyl rhenium(I) (MEA39).

According to general procedure **C** from (*S*)-2-amino-3-[1-(2-{2-[2-(2-{4-[2,4,6-trioxo-5-(4-phenoxyphenyl)hexahydropyrimidin-5-yl]piperazine-1‑yl}ethoxy)ethoxy]-ethoxy}ethyl)-*1H*-1,2,3-triazol-4-yl]propionic acid potassium salt (**14**) (100 mg, 0.136 mmol, 1 eq.). Purification in portions by HPLC system A yielded the product as a white solid. **Yield:** 23.7 mg (0.025 mmol, 18%). **^1^H-NMR** (600 MHz, CD_3_OD): δ (ppm) 2.89 (dd, *J*= 29.2 Hz, *J* = 11.3 Hz, 2H), 3.12 (t, *J* = 8.4 Hz, 2H), 3.49 (d, *J* = 11.3 Hz, 2H), 3.51-3.62 (m, 8H), 3.76-3.80 (m, 2H), 3.84 (ddd, *J* = 11.3 Hz, *J*= 6.5 Hz, *J* = 3.2 Hz, 1H), 3.91 (ddd, *J* = 11.3 Hz, *J*= 7.0 Hz, *J* = 3.2 Hz, 1H), 4.12 (ddd, *J* = 5.9 Hz, *J*= 4.5 Hz, *J* = 3.2 Hz, 1H), 4.55 (ddd, *J* = 14.3 Hz, *J*= 6.5 Hz, *J* = 3.2 Hz, 1H), 4.69 (ddd, *J* = 14.3 Hz, *J*= 6.5 Hz, *J* = 3.2 Hz, 1H), 5.22 (d, *J* = 11.3 Hz, 1H), 5.86 (dd, *J*= 11.3 Hz, *J* = 6.0 Hz, 1H), 7.01-7.07 (m, 4H), 7.19 (td, *J*= 7.2 Hz, *J* = 1.1 Hz, 1H), 7.38-7.43 (m, 2H), 7.49 (d, *J* = 8.8 Hz, 2H), 8.02 (s, 1H). **^13^C-NMR** (150 MHz, CD_3_OD): δ (ppm) 46.0, 46.1, 52.5, 53.0, 54.1, 54.3, 65.2, 69.5, 71.1, 71.2, 71.4, 71.5, 76.2, 119.5, 120.9, 125.5, 127.2, 129.5, 130.6, 131.2, 143.5, 150.5, 157.3, 160.7, 171.5, 171.6, 184.9, 196.9, 197.4, 198.2. **MS-ESI(+):** *m/z* = 987.2295 (M + Na)^+^, 956.2476 (M + H)^+^.


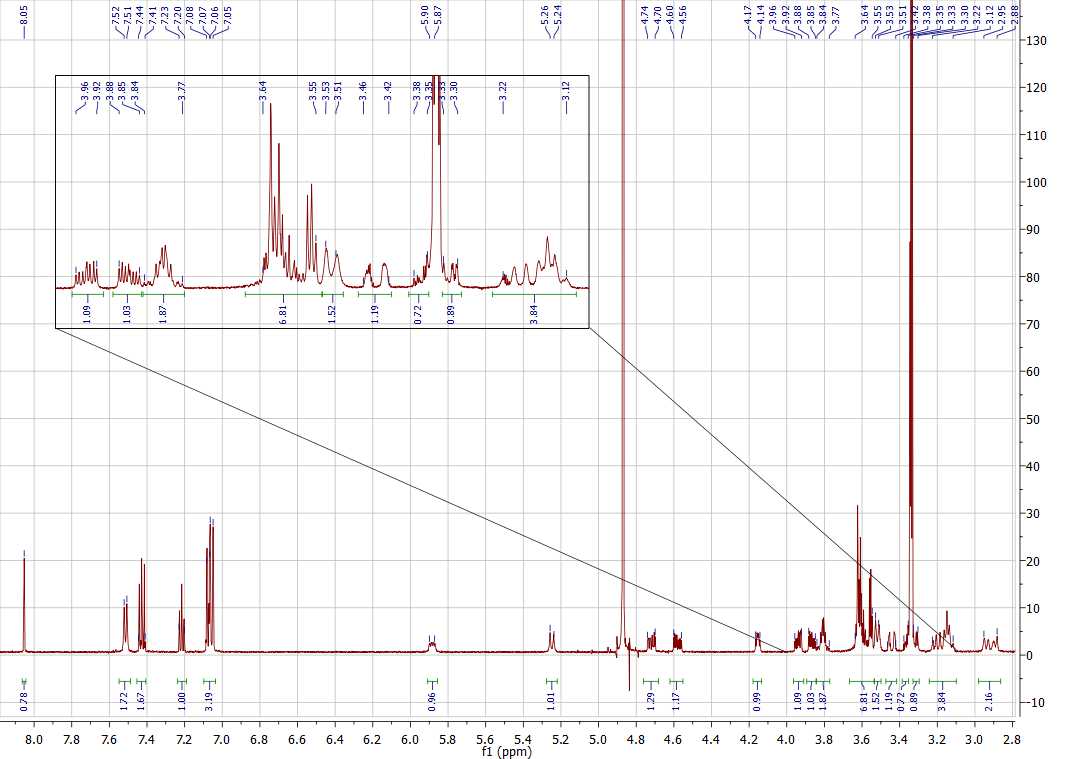


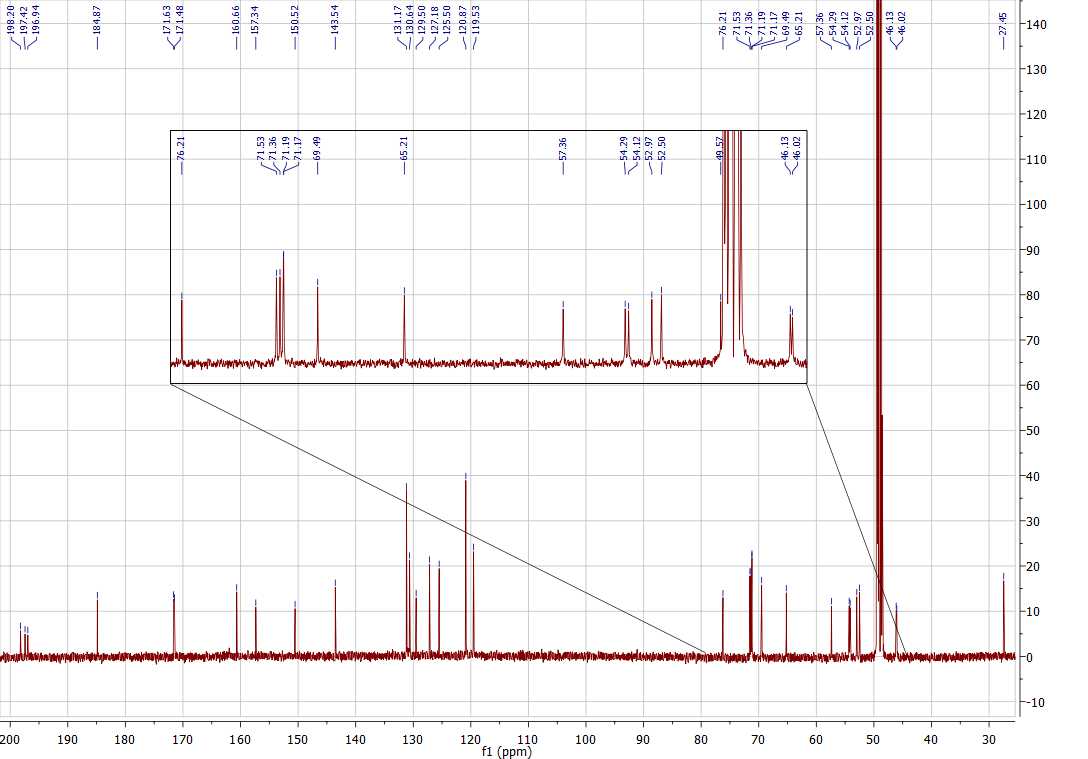


### Synthesis and spectroscopic data of the lysine based barbiturate

#### Methyl *N*^2^-(*tert*-butoxycarbonyl)-*N*^6^-(2,2,2-trifluoroacetyl)-*N*^2^-{[1-(2-{2-[2-(2-{4-[2,4,6-trioxo-5-(4-phenoxyphenyl)hexahydropyrimidin-5-yl]piperazin-1-yl}ethoxy)ethoxy]ethoxy}ethyl)-1*H*-1,2,3-triazol-4-yl]methyl}-L-lysinate (15).

According to general procedure **A** from (*S*)-methyl 2-[(*tert*-butoxycarbonyl)(prop-2-yn-1-yl)amino]-6-(2,2,2-trifluoroacetamido)hexanoate (**8**) (50 mg, 0.127 mmol, 1 eq.). The product was directly used in the next step. **Yield:** 59 mg (0.061 mmol, 48%). **MS-ESI(+):** *m/z* = 998.4203 (M + Na)^+^, 976.4389 (M + H)^+^.

#### Methyl *N*^6^-(2,2,2-trifluoroacetyl)-*N*^2^-{[1-(2-{2-[2-(2-{4-[2,4,6-trioxo-5-(4-phenoxyphenyl)hexahydropyrimidin-5-yl]piperazin-1-yl}ethoxy)ethoxy]ethoxy}ethyl)-1*H*-1,2,3-triazol-4-yl]methyl}-L-lysinate TFA salt (16).

According to general procedure **B** from methyl *N*^2^-(*tert*-butoxycarbonyl)-*N*^6^-(2,2,2-trifluoroacetyl)-*N*^2^-{[1-(2-{2-[2-(2-{4-[2,4,6-trioxo-5-(4-phenoxyphenyl)hexahydropyrimidin-5-yl]piperazin-1-yl}ethoxy)ethoxy]ethoxy}ethyl)-1*H*-1,2,3-triazol-4-yl]methyl}-L-lysinate (**15**) (654 mg, 0.67 mmol, 1 eq.). The product was directly used for the next step. **Yield:** 420 mg (0.42 mmol, 63%). **MS-ESI(+):** *m/z* = 898.3652 (M + Na)^+^, 876.3862 (M + H)^+^.

#### {[1-(2-{2-[2-(2-{4-[2,4,6-Trioxo-5-(4-phenoxyphenyl)hexahydropyrimidin-5-yl]piperazin-1-yl}ethoxy)ethoxy]ethoxy}ethyl)-1*H*-1,2,3-triazol-4-yl]methyl}-L-lysinate (17).

Methyl *N*^6^-(2,2,2-trifluoroacetyl)-*N*^2^-{[1-(2-{2-[2-(2-{4-[2,4,6-trioxo-5-(4-phenoxyphenyl)hexahydropyrimidin-5-yl]piperazin-1-yl}ethoxy)ethoxy]ethoxy}ethyl)-1*H*-1,2,3-triazol-4-yl]methyl}-L-lysinate TFA salt (**16**) (244 mg, 0.25 mmol, 1 eq.) was dissolved in MeOH (3 mL) and a solution of potassium carbonate (0.85 g, 6.14 mmol, 25 eq.) in water (2 mL) was added. The obtained solution was stirred at 75 °C for 3 h. The mixture was neutralized with trifluoroacetic acid (pH = 7). The solvent was removed under reduced pressure until a slight turbidity was formed. Water (1 mL) was added and the crude product was purified in portions by HPLC system A (without TFA in the solvent system), yielding the product as a sticky yellow oil. **Yield:** 151 mg (0.20 mmol, 79%). **^1^H-NMR** (600 MHz, CD_3_OD): δ (ppm) = 1.48-1.66 (m, 2H), 1.72 (p, *J* = 7.7 Hz, 2H), 1.95-2.10 (m, 2H), 2.94 (t, *J* = 7.7 Hz, 4H), 3.21 (q, *J* = 7.3 Hz, 2H), 3.34-3.38 (m, 2H), 3.58-3.61 (m, 8H), 3.62-3.65 (m, 2H), 3.79-3.82 (m, 2H), 3.88 (t, *J* = 5.1 Hz, 2H), 3.97 (dd, *J* = 7.3 Hz, *J* = 4.9 Hz, 1H), 4.38-4.46 (m, 2H), 4.60 (t, *J* = 5.1 Hz, 2H), 7.00-7.06 (m, 4H), 7.18 (td, *J* = 7.4 Hz, *J* = 1.1 Hz, 1H), 7.37-7.41 (m, 2H), 7.48 (d, *J* = 8.5 Hz, 2H), 8.15 (s, 1H). **^13^C-NMR** (150 MHz, CD_3_OD): δ (ppm) = 22.9, 28.0, 29.9, 40.2, 41.8, 46.1, 51.5, 57.3, 60.0, 65.2, 70.2, 71.1, 71.3, 71.3, 71.3, 76.2, 119.5, 120.9, 125.5, 127.4, 129.5, 130.6, 131.2, 150.6, 157.3, 160.6, 161.8, 162.3, 170.9, 171.5. **MS-ESI(+):** *m/z* = 766.3863 (M + H)^+^.


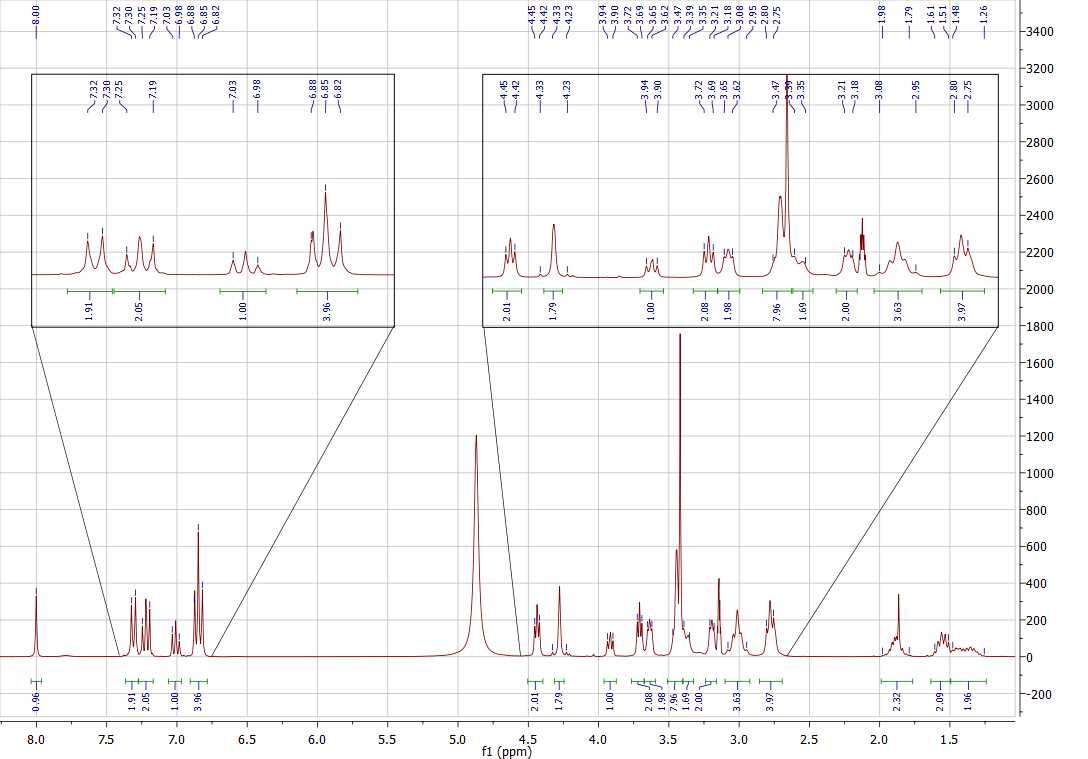


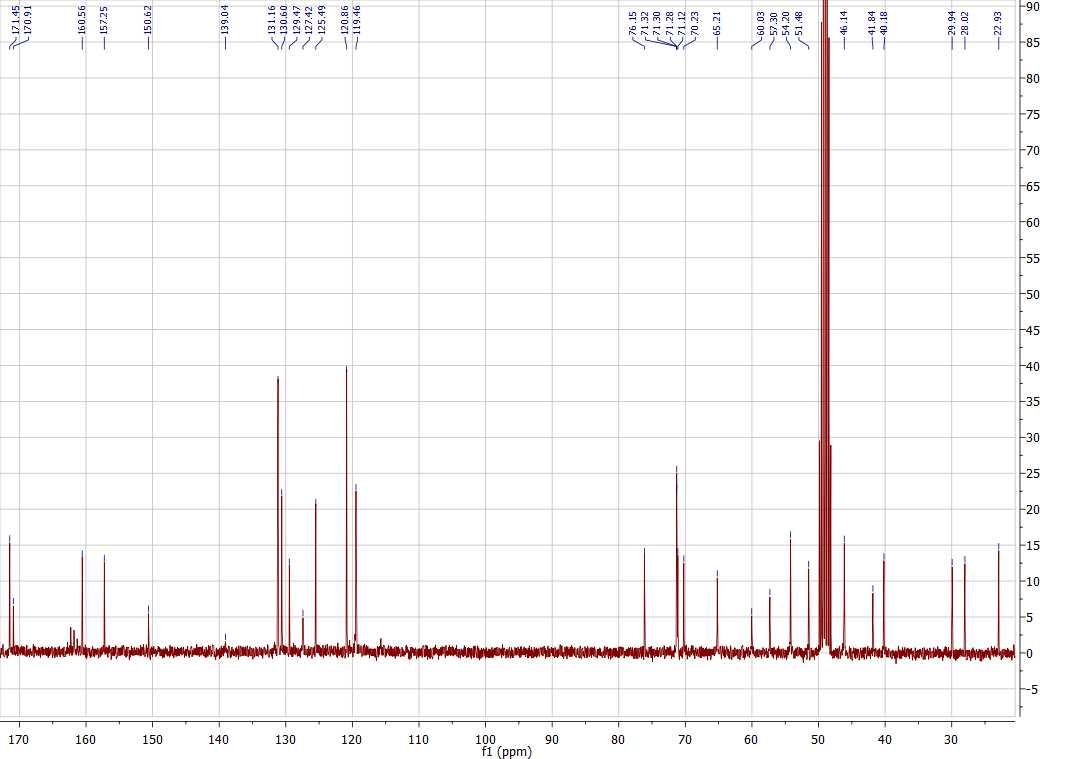


#### {[1-(2-{2-[2-(2-{4-[2,4,6-Trioxo-5-(4-phenoxyphenyl)hexahydropyrimidin-5-yl]piperazin-1-yl}ethoxy)ethoxy]ethoxy}ethyl)-1*H*-1,2,3-triazol-4-yl]methyl}-L-lysinate tricarbonylrhenium(I) (MEA61).

According to general procedure **C** from {[1-(2-{2-[2-(2-{4-[2,4,6-trioxo-5-(4-phenoxyphenyl)hexahydropyrimidin-5-yl]piperazin-1-yl}ethoxy)ethoxy]ethoxy}ethyl)-1*H*-1,2,3-triazol-4-yl]methyl}-L-lysinate (**17**) (47 mg, 0.061 mmol, 1 eq.). Purification in portions by HPLC system A yielded the product as a white solid. **Yield:** 25.8 mg (0.025 mmol, 41%). **^1^H-NMR** (600 MHz, CD_3_OD): δ (ppm) 1.58-1.80 (m, 4H), 1.94 (q, *J* = 7.4 Hz, 2H), 2.90-3.00 (m, 4H), 3.11-3.22 (m, 4H), 3.32-3.36 (m, 2H), 3.49-3.55 (m, 2H), 3.57-3.67 (m, 8H), 3.80 (t, *J* = 5.0 Hz, 2H), 3.92 (t, *J* = 5.0 Hz, 2H), 4.27 (dd, *J* = 15.7 Hz, *J*= 5.4 Hz, 1H), 4.36 (d, *J* = 15.7 Hz, 1H), 4.66 (q, *J* = 4.4 Hz, 2H), 6.71 (d, *J* = 5.4 Hz, 1H), 6.99-7.06 (m, 4H), 7.18 (t, *J*= 7.4 Hz, 1H), 7.40 (t, *J*= 8.0 Hz, 2H), 7.48 (d, *J* = 8.6 Hz, 2H), 8.17 (s, 1H). **^13^C-NMR** (150 MHz, CD_3_OD): δ (ppm) 24.0, 28.3, 28.3, 33.6, 33.7, 40.4, 46.1, 49.0, 53.0, 53.8, 54.2, 57.3, 65.2, 67.5, 69.8, 71.2, 71.3, 71.4, 71.5, 76.2, 119.5, 120.9, 124.9, 125.5, 129.5, 130.6, 131.2, 149.0, 150.5, 157.3, 160.7, 171.5, 185.8, 196.6, 196.7, 198.4. **MS-ESI(+):** *m/z* = 1058.3031 (M + Na)^+^, 1036.3211 (M + H)^+^.

**
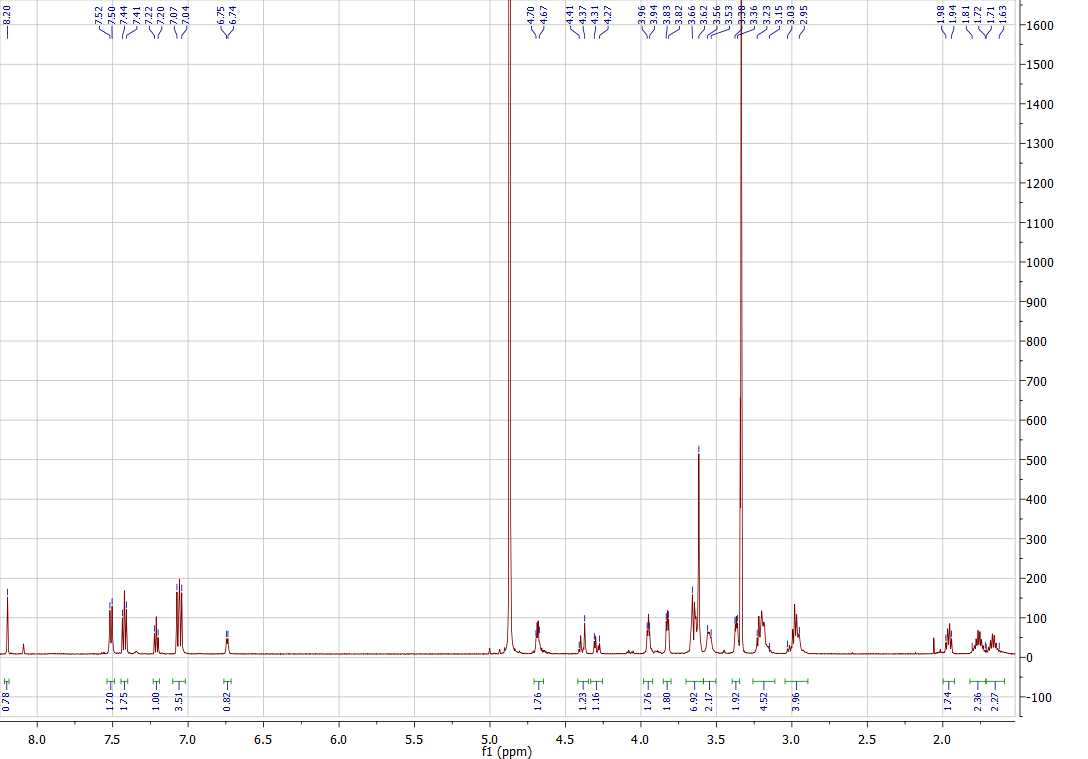
**

**
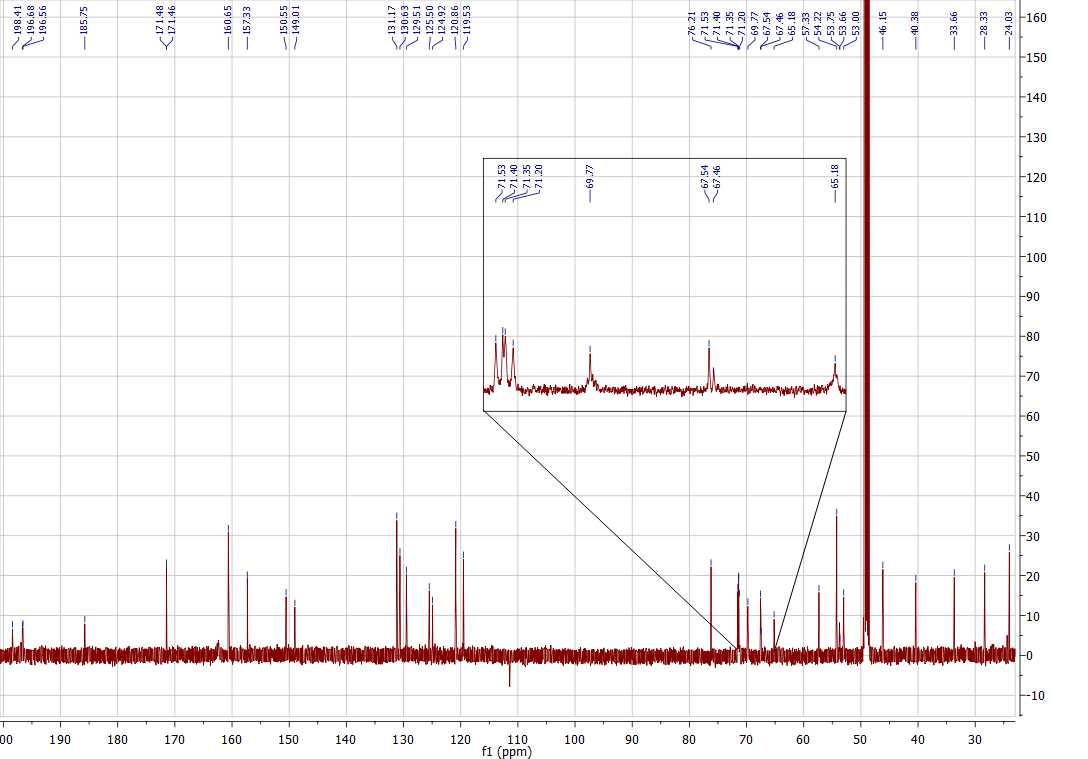
**

### Synthesis and spectroscopic data of the HYNIC precursor (19)

#### *tert*-Butyl-2-{5-[(2-{2-[2-(2-{4-[2,4,6-trioxo-5-(4-phenoxyphenyl)hexahydropyrimidin-5-yl]piperazine-1-yl}ethoxy)ethoxy]ethoxy}ethyl)carbamoyl]pyridine-2-yl}hydrazine-1-carboxylate (18)

Triethylamine (0.75 mL, 5.40 mmol, 3 eq.) was added to a solution of 5-[4-(2-{2-[2-(2-aminoethoxy)ethoxy]ethoxy}ethyl)piperazine-1-yl]-5-(4-phenoxyphenyl)-pyrimidine-2,4,6-trione (**12**) (1.00 g, 1.80 mmol, 1 eq.) and 2,5‑dioxopyrrolidin-1-yl-6-[2-(*tert*-butoxycarbonyl)hydrazinyl]nicotinate (1.89 g, 5.40 mmol, 3 eq.) in tetrahydrofuran (50 mL). The mixture was stirred at room temperature for 12 h and the solvent was removed under reduced pressure. The crude product was purified by column chromatography on deactivated silica (pretreatment with 2.5% triethylamine in ethyl acetate:methanol 4:1, solvent: ethyl acetate:methanol 4:1). The product was isolated as a white solid. **Yield:** 1.13 g (1.43 mmol, 79%). **^1^H-NMR** (400 MHz, CDCl_3_): δ = 1.41 (s, 9H), 2.16 (s, 2H), 2.60 (s, 1H), 2.67-2.83 (m, 9H), 3.49-3.67 (m, 15H), 6.66 (d, *J* = 8.6 Hz, 1H), 6.92 (d, *J* = 9.0 Hz, 2H), 6.96-7.01 (m, 2H), 7.11 (t, *J* = 7.4 Hz, 1H), 7.28-7.35 (m, 2H), 7.44 (d, *J* = 8.4 Hz, 2H), 7.61 (s, 1H), 7.80 (s, 1H), 8.04 (d, *J* = 8.4 Hz, 1H), 8.58 (s, 1H). **^13^C-NMR** (100 MHz, CDCl_3_): δ = 28.3, 39.8, 46.8, 53.5, 57.1, 60.5, 67.5, 70.1, 70.2, 70.3, 70.5, 74.4, 81.5, 105.9, 118.4, 119.7, 122.3, 124.1, 128.7, 130.0, 138.8, 146.5, 150.6, 156.1, 156.3, 158.4, 161.7, 165.9, 170.7, 174.5. **MS-ESI(+):** *m/z* = 791.3700 [M+H]^+^, 813.3517 [M+Na]^+^.

#### 6-Hydrazinyl-N-(2-{2-[2-(2-{4-[2,4,6-trioxo-5-(4-phenoxyphenyl)hexahydro-pyrimidin-5-yl]piperazin-1-yl}ethoxy)ethoxy]ethoxy}ethyl)nicotinoamide (19)

A solution of hydrogen chloride in dioxane (4M, 1.28 mL, 5.12 mmol, 1.3 eq.) was added to a solution of *tert*-Butyl-2-{5-[(2-{2-[2-(2-{4-[2,4,6-trioxo-5-(4-phenoxyphenyl)hexa-hydro-pyrimidin-5-yl]piperazine-1-yl}ethoxy)ethoxy]ethoxy}ethyl)carbamoyl]pyridine-2-yl}-hydrazine-1-carboxylate (**18**) (440 mg, 3.90 mmol, 1 eq.) in tetrahydrofuran:methanol (3:1, 12 mL) at 0 °C. The mixture was stirred at room temperature for 4 d and the solvent was removed under reduced pressure. The crude product was purified by column chromatography (solvent: ethyl acetate:methanol 1:2). The product was isolated as a white solid. **Yield:** 484 mg (0.7 mmol, 18%). **^1^H-NMR** (600 MHz, CD_3_OD): δ = [1.64-1.69 (m, 1H), 1.79-1.86 (m, 1H), 2.94 (d, *J* = 11.7 Hz, 2H), signals in brackets correspond to different HCl-salts], 3.14-3.25 (m, 4H), 3.37 (t, *J* = 4.9 Hz, 2H), 3.53-3.61 (m, 6H), 3.62-3.67 (m, 10H), 3.81-3.84 (m, 2H), 6.99-7.05 (m, 5H), 7.17 (td, *J* = 7.4 Hz, *J* = 1.1 Hz, 1H), 7.36-7.41 (m, 2H), 7.48 (d, *J* = 8.4 Hz, 2H), 8.22 (dd, *J* = 9.3 Hz, *J* = 2.0 Hz, 1H), 8.46 (d, *J* = 2.0 Hz, 1H). **^13^C-NMR** (150 MHz, CD_3_OD): δ = 40.9, 45.6, 46.2, 54.3, 57.4, 62.1, 65.3, 68.1, 70.4, 71.2, 71.3, 71.5, 76.2, 111.9, 119.5, 120.8, 121.9, 125.5, 129.5, 130.6, 131.2, 139.8, 141.1, 150.5, 157.2, 157.3, 160.6, 165.8, 171.4. **MS-ESI(+):** *m/z* = 691.3089 [M+H]^+^, 713.3000 [M+Na]^+^.


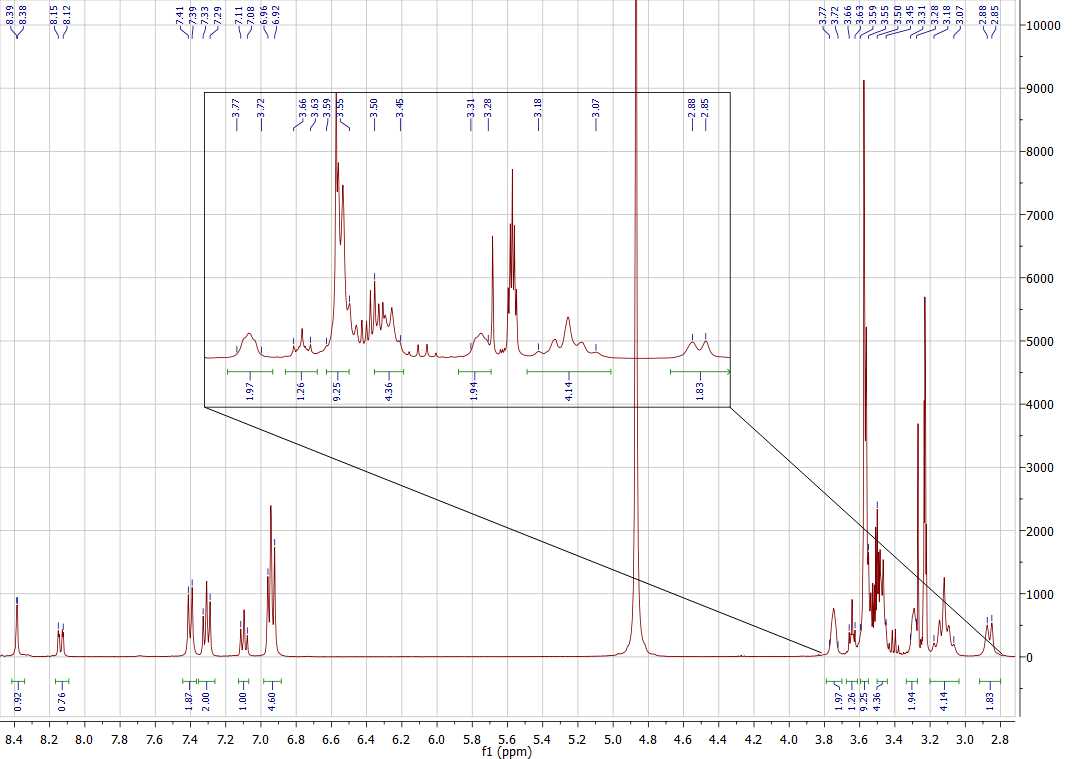


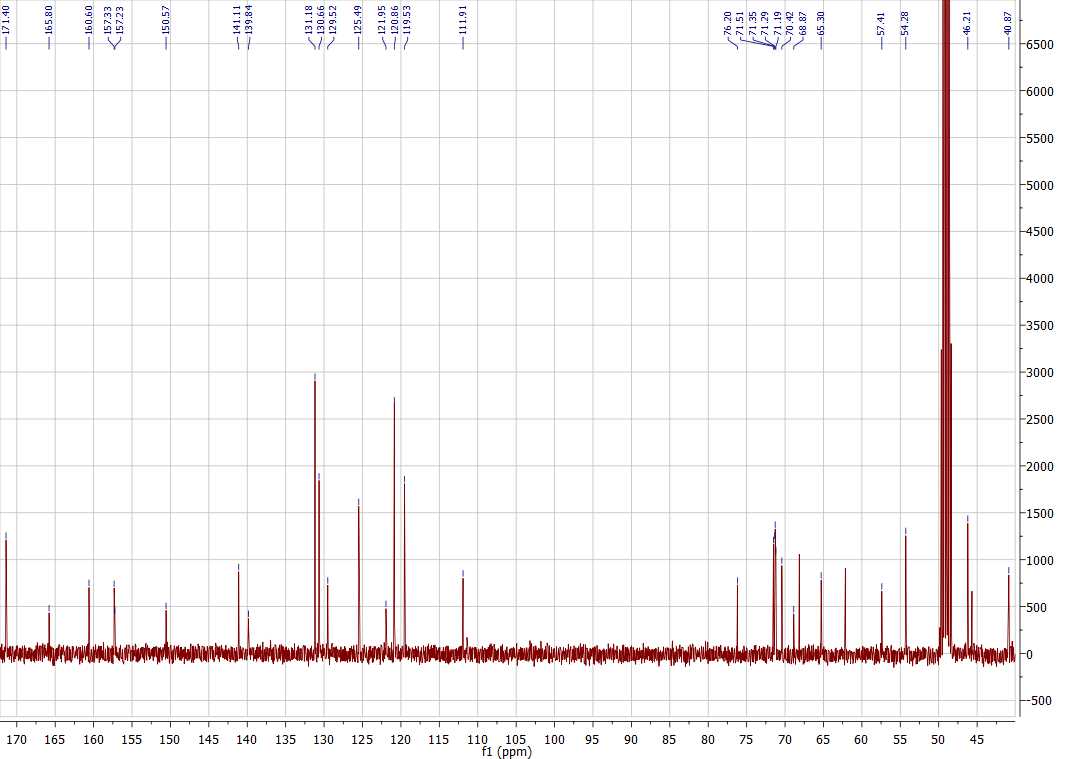


## Radiosynthesis, *in vitro* data

### General considerations

All *chemicals*, *reagents*, and *solvents* for the radiosynthesis of tracers were analytical grade, purchased from commercial sources and used without further purification unless otherwise specified. Only solvents of pharmaceutical purity from ABX and Milli-Q^®^-water or water for injection from B. Braun were used for radiosynthesis.

Sep-Pak^®^ C-18 Plus cartridges from Waters were used for the *purification* of radiolabeled compounds. The cartriges were conditioned with ethanol (5 mL) followed by water (10 mL) prior to use.

In some cases vessels were *coated on the surface* using Sigmacote^®^ from Sigma-Aldrich.

*[^99m^Tc]Pertechnetate* for labeling was eluted from a ^99^Mo/^99m^Tc-mother-daughter-nuclide generator *Elumatic III* from Iba Molecular using 0.9% saline.

*Identification* of labeled compounds was performed by co-injection of a non-radioactive Rhenium-based reference compound on HPLC system C if possible. In the case of the HYNIC derivative **[^99m^Tc]MEA223** a reference compound could not be synthesized, the identification was done by mass spectrometry of the decay product **[^99g^Tc]MEA223**.

*Radiochemical yields* are based on the sodium [^99m^Tc]pertechnetate initial activity and are decay corrected.

*Radiochemical purity* is the ratio of the fraction of product radioactivity and total radioactivity and was determined by analytical HPLC system C.

The *time of radiosynthesis* is given as the time between application of sodium [^99m^Tc] pertechnetate to the kit for synthesis and reconstitution of the tracer for application.

*Measurements of total radioactivity* for the determination of experimental log*D*-values were performed using a gamma-counter Wallac Wizard 3‘‘ from PerkinElmer Life Science.

*General measurements of radioactivity* were done on an *Isomed 2010-*activimeter from Med Nuklearmedizintechnik.

To *heat up kits for radiolabeling* a DRI-Block^®^ DB-3D-heating block from Techne, Bibby Scientific was used.

*Osmolalities* of radiolabeled compounds for animal experiments were determined with a 15 µL sample on an Osmomat 030 osmometer from Gonotec.

*Zentrifugation* was done in Eppendorf vessels (1.5 mL) in a MCF-2360 zentrifuge from **Lms Consult GmbH & Co. KG.**

***Incubation* of samples for the determination of *serum stability* was performed using a** PST-60 HL plus thermo shaker from **Kisker Biotech GmbH & Co. KG.**

*Separation*, *purification* and determination of *(radio)chemical purity* of the compounds were performed by using the preparative, semipreparative and analytical reversed-phase HPLC systems A, B and C as already stated under “1.1. General considerations”.

Following a method described by Prante *et al.*[13], the *distribution coefficient (logD_exp_)* of the radioactive compound was determined in a two-phase system consisting of 1-octanol and PBS-buffer (pH = 7.4) to determine the lipophilicity. For this purpose, the corresponding ligand (~ 20 kBq) was dissolved in buffer (500 μL). 1-Octanol (500 μL) was added and the mixture was shaken at room temperature for 1 min. To achieve phase separation, the system was centrifuged for 2 min at 3000 rpm. Subsequently a part of the octanol phase (400 μL) was removed and buffer (400 μL) was added. The combined phases were shaken and layers were separated according to the description above. Aliquots of the buffer and octanol layers (3 × 100 μL from every layer) were taken to measure radioactivity in a γ-counter. The measurement provided the activity in counts per minute (cpm), the values were decay corrected. By calculating the quotient of $\frac{cpm (1-Octanol)}{cpm (PBS)}$ the *logD_exp_* can be determined as $\log\frac{cpm (1-Octanol)}{cpm (PBS)}$.

The *serum stability* of radioactive compounds was evaluated by incubation in human and murine serum at 37 °C for up to 120 min. An aliquot of radioactive product (20 μL, ~5 MBq) in PBS-buffer was added to a sample of serum (200 μL), and the mixture was incubated at 37 °C. Samples of 20 μL each were taken after periods of 10, 30, 60, 90 and 120 min and quenched in MeOH/CH_2_Cl_2_ (1:1 (v/v), 100 μL) followed by centrifugation for 2 min. The clear solution was analyzed by analytical radio-HPLC system C.

To determine the relative *potency of new inhibitors* in terms of *IC_50_-values* the method developed by Huang *et al*. was applied in an *in vitro* inhibition study.[14] Compounds of interest were evaluated regarding affinity to MMP-2, -8, -9, -13 and -14 using the artificial MMP-substrate (7-Methoxycoumarine-4-yl)-acetyl-Pro-Leu-Gly-Leu-[3‑(2,4-dinitrophenyl)-l-2,3-diaminopropionyl]-Ala-Arg-NH_2_ (R&D Systems, Minneapolis, USA). The activated form of the corresponding MMPs (2 nM each) were incubated at 37 °C with the barbiturate derivatives in different concentrations (10 pM-1 mM) in TRIS-buffer (50 mM, pH = 7.5), sodium chloride (0.2M), calcium chloride (5 mM), zinc sulfate (20 µM) and Brij® (0.05%) for 30 min. Samples of this mixture (90 µL) were added to the substrate (50 µM, 10 µL) at 37 °C and the increase of fluorescence as a function of time was measured using a TriStar² LB 942 Analyzers (Berthold Technologies, λ_Ex_ = 330 nm, λ_Em_ = 390 nm). The linear change of relative fluorescence after 10 min was plotted as a function of the concentration of the used inbhibitor. The IC_50_-values were determined by non-linear regression using the Grace 5.1.8 software (Linux).

To perform the mass spectrometry the ^99m^Tc-labeled compounds were stored under nitrogen atmosphere at -20°C for about 60 h, until measureable radioactivity was below the permitted limit for transportation and handling in a non-(radiation)-controlled area. The sample was diluted with acetonitrile (1:5).

All *animal experiments* were conducted in accordance with local institutional guidelines for the care and use of laboratory animals.

### ^99m^Tc-labeling using „kits“

The labeling of compounds was accomplished by using kits. A kit is a sterile glas bottle which is closed by a septum, it contains all reagents necessary for the synthesis and a protective gas (argon).[15, 16] The reagents are adapted to the precursors and the field of application. In this work, two different kits for radiosynthesis were used. One is for generating a triaquatricarbonyl-complex [^99m^Tc(CO)_3_(OH_2_)_3_]^+^ which is used for labeling, the other one is for the labeling of HYNIC-derivatives and contains all co-ligands. Both kits were prepared in-house.

#### [^99m^Tc(CO)_3_(OH_2_)_3_]^+^- kit for radiosynthesis

Preparation of the [^99m^Tc(CO)_3_(OH_2_)_3_]^+^ - kit was done by mixing K_2_[H_3_BCO_2_] (4.5 mg, 0.033 mmol), potassium sodium tartrate dihydrate (7.0 mg, 0.028 mmol) and sodium borate decahydrate (7.0 mg, 0.018 mmol) in a glass bottle. The bottle was capped under argon with a septum and stored at -20 °C until use.

##### Synthesis of K_2_[H_3_BCO_2_]

Carbon monoxide was slowly bubbled through a solution of BH_3_ ^.^ THF (1M, 30 mL). The gas stream was passed through a cooling trap adjusted to -44 °C and then bubbled through a solution of potassium hydroxide (5.00 g, 89.1 mmol) in ethanol (200 mL) in a Schlenk tube which was cooled to -78 °C. After 2 h the Schlenk tube was disconnected and heated to reflux for 45 min. After cooling to room temperature, the precipitate was filtered, washed with cold ethanol (2 × 5 mL) and dried. Yield**:** 1.10 g (8.1 mmol, 27%; Lit.[15]: 43%)

##### Generating the [^99m^Tc(CO)_3_(OH_2_)_3_]^+^-complex for labeling

The kit for radiosynthesis described under “1.3.2.1 [^99m^Tc(CO)_3_(OH_2_)_3_]^+^- kit for radiosynthesis” was warmed to room temperature and sodium [^99m^Tc]pertechnetat (1-2 mL) from a generator was added. The bottle was carefully slewed to dissolve all reactants and heated to 100 °C for 20 min. Occasionally quality control by HPLC was performed, but generally the complex was directly used for the labeling of precursors. Radio-HPLC (HPLC System A): *t*_R_ = 19.4 min.; rcp (HPLC System A): > 99%.

#### HYNIC-kit for radiosynthesis

Preparation of the HYNIC - kit was done by mixing 3,3′,3″-phosphanetriyltris(benzenesulfonic acid) trisodium salt (TPPTS, 7 mg, 12 µmol), tricine (6.5 mg, 36 µmol), mannitol (40 mg, 200 µmol), disodium succinate hexahydrate (38.5 mg, 140 µmol) and succinic acid (12.7 mg, 108 µmol) in a glass bottle.[17] The bottle was capped under argon with a septum and stored at -20 °C until use. For labeling of suitable precursors the bottle was warmed to room temperature and sodium [^99m^Tc]pertechnetat (1-2 mL) from a generator was added. The kit was directly used for labeling.

### General procedure AR for the ^99m^Tc-labeling of barbiturates

The precursor for labeling was dissolved (see solvent and volume at the corresponding experiment) and added to the corresponding kit for radiosynthesis (Figure 2: Preparation of ^99m^Tc-based radiopharmaceuticals. The solution was heated to 100 °C for 16-20 min. After cooling to room temperature, the crude reaction mixture was purified by HPLC system B. Products were collected in vessels which were coated on the surface using Sigmacote^®^. The temperature sensitive products were diluted with water (15 mL) and filtered through a Sep-Pak^®^ C-18 Plus cartridge. The products were re-eluted with ethanol (8 mL) and collected in vessels which were coated on the surface using Sigmacote^®^. Solvent was removed under reduced pressure while not heating the radiolabeled compounds. The residues were solved in isotonic sodium chloride solution containing 1.6 vol% of Tween80^®^ (100-500 µL). To determine the radiochemical purity (RCR) a part of the solution (20 µL) was analyzed by HPLC System C. Further parameters (pH, osmolality) were also determined from the formulated solution at random.


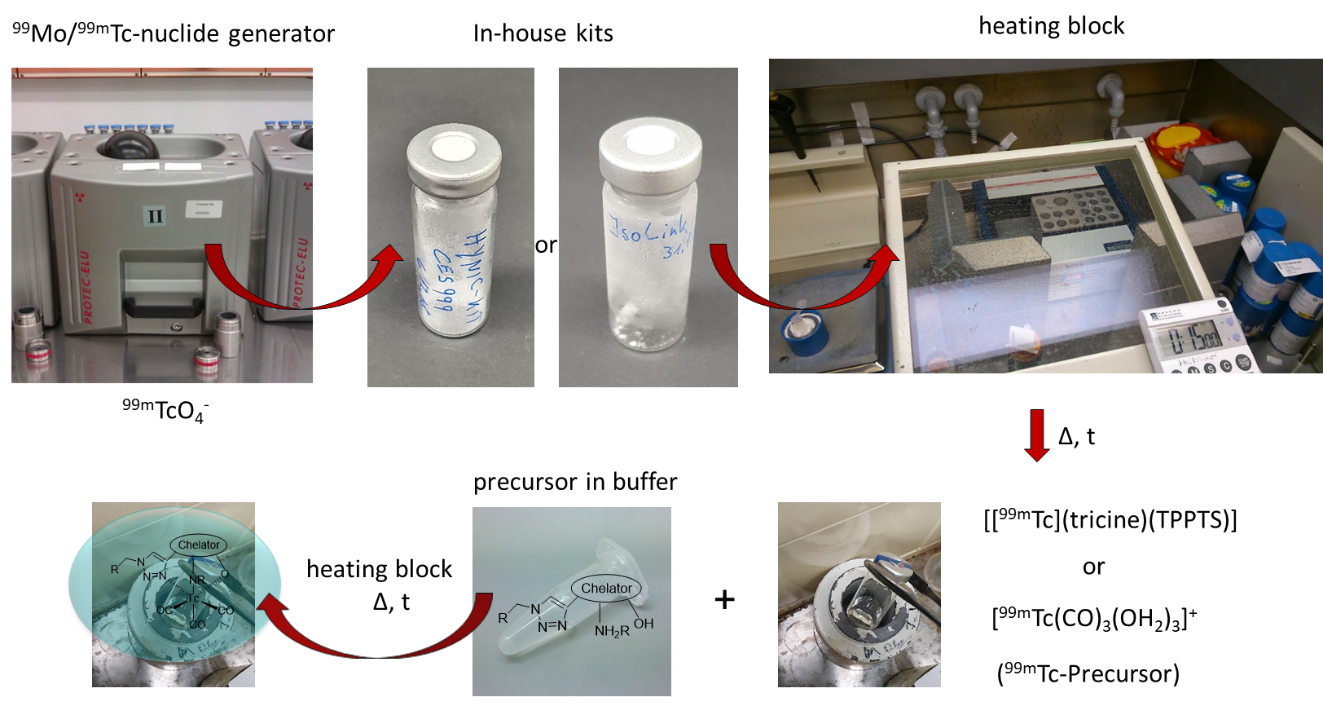


Figure 2: Preparation of ^99m^Tc-based radiopharmaceuticals

### Glycine based barbiturate tracer [^99m^Tc]MEA39

The synthesis of the glycine-based tracer **[^99m^Tc]MEA39** is derived from the “click-to-chelate” concept which was developed by Mindt *et al*.[18] Since problems occurred applying the concept on the barbiturate-based compounds directly, the precursors were isolated after the “click”-reaction and were then used for ^99m^Tc-labeling after deprotection. The [^99m^Tc(CO)_3_(OH_2_)_3_]^+^ - kit was used to label *(S*)-2-amino-3-[1-(2-{2-[2-(2-{4-[2,4,6-trioxo-5-(4-phenoxyphenyl)-hexahydropyrimidin-5-yl]piperazine-1‑yl}ethoxy)ethoxy]-ethoxy}ethyl)-*1H*-1,2,3-triazol-4-yl]propionic acid TFA salt (**14**, 1 mg in 500 µL PBS) according to general procedure AR. The mixture was heated for 16 min and the product was isolated by radio-HPLC B (Figure 3). Used ^99m^Tc as pertechnetate: 2485-5688 MBq; rcy (d.c.): 21% ± 11% (n = 6); rcp: > 99%; time for synthesis: 131 min ± 9 min (n = 6); t*_R_* (HPLC C) = 8.6 min.

Figure 3: Radiosynthesis of the glycine based barbiturate tracer [^99m^Tc]MEA39

Quality control was performed by analytical radio-HPLC C (Figure 4).


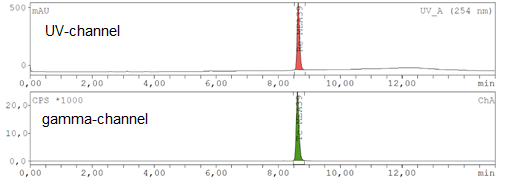


Figure 4: Quality control of the glycine based barbiturate tracer [^99m^Tc]MEA39; performed by co-injection of the corresponding Re-compound MEA39

Additional quality control was performed by MS analysis. The spectrum is in accordance with the expected compound (Figure 5).

.^
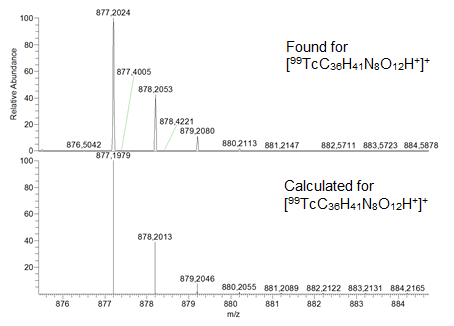
^

Figure 5: Orbitrap MS-spectra of compound [^99^Tc]MEA39

To evaluate the biological activity of the inhibitor in terms of potency and selectivity, an *in vitro* inhibition assay was performed using the chelator **14** as well as the reference Re-compound **MEA39**. The results are summarized in Table 2.

Table 2: Data of the *in vitro* assay for the glycine based compounds

| Compound | MMP-2  [nM] | MMP-8  [nM] | MMP-9  [nM] | MMP-13  [nM] | MMP-14  [nM] |
| --- | --- | --- | --- | --- | --- |
| 14 | 0.6 ± 0.1 | 3.6 ± 2.6 | 1.4 ± 0.3 | 27 ± 9 | 1.5 ± 0.4 |
| MEA39 | 2 ± 0.5 | 28 ± 7 | 0.9 ± 0.07 | 2 ± 0.3 | 5.9 ± 0.65 |

To exclude major stability issues, the compound **[^99m^Tc]MEA39** was incubated with samples of human and murine blood serum (Figure 6 + Figure 7). The tracer showed no decomposition using the described procedure.


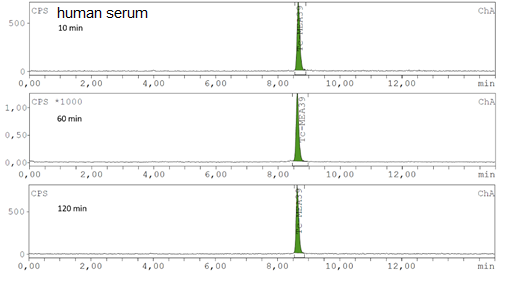


Figure 6: Evaluation of the stability of compound [^99m^Tc]MEA39 in human blood serum


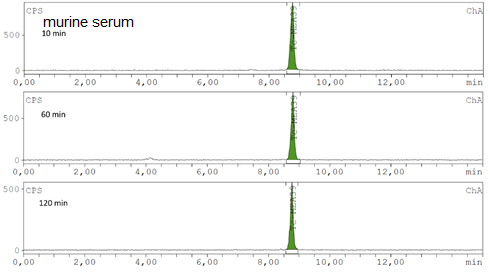


Figure 7: Evaluation of the stability of compound [^99m^Tc]MEA39 in murine blood serum

### Lysine based barbiturate tracer [^99m^Tc]MEA61

The synthesis of the lysine-based tracer **[^99m^Tc]MEA61** bis derived from the “click-to-chelate” concept which was developed by Mindt *et al*.[18] Since problems occurred applying the concept on the barbiturate-based compounds directly, the precursors were isolated after the “click”-reaction and were then used for ^99m^Tc-labeling after deprotection. The [^99m^Tc(CO)_3_(OH_2_)_3_]^+^ - kit was used to label {[1-(2-{2-[2-(2-{4-[2,4,6-trioxo-5-(4-phenoxyphenyl)hexahydropyrimidin-5-yl]piperazin-1-yl}ethoxy)ethoxy]ethoxy}ethyl)-1*H*-1,2,3-triazol-4-yl]methyl}-L-lysinate di TFA salt (**17**, 1 mg in 500 µL PBS) according to general procedure AR. The mixture was heated for 16 min and the product was isolated by radio-HPLC B (Figure 8). Used ^99m^Tc as pertechnetate: 3772 – 6208 MBq; rcy (d.c.): 23% ± 11% (n = 3); rcp: > 99%; Time for synthesis: 130 min ± 53 min (n = 3); t_R_ (HPLC C) = 7.9 min.

Figure 8: Radiosynthesis of the lysine based barbiturate tracer [^99m^Tc]MEA61

Quality control was performed by analytical radio-HPLC C (Figure 9).


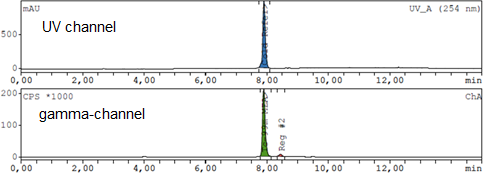


Figure 9: Quality control of the lysine based barbiturate tracer [^99m^Tc]MEA61; performed by co-injection of the corresponding Re-compound MEA61

Additional quality control was performed applying mass spectrometry. The spectrum is in accordance with the expected compound (Figure 10).


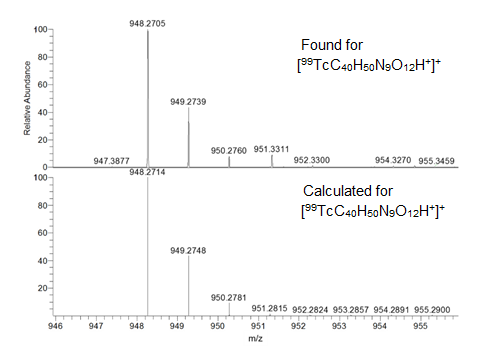


Figure 10: Orbitrap MS-spectra of compound [^99^Tc]MEA61

To evaluate the biological activity of the inhibitor in terms of potency and selectivity, an *in vitro* inhibition assay was performed using the chelator **17** as well as the reference Re-compound **MEA61**. The results are summarized in Table 3.

Table 3: Data of the *in vitro* assay for the lysine based compounds

| compound | MMP-2  [nM] | MMP-8  [nM] | MMP-9  [nM] | MMP-13  [nM] | MMP-14  [nM] |
| --- | --- | --- | --- | --- | --- |
| 17 | 7 ± 3 | 18 ± 3 | 10 ± 2 | 58 ± 9 | 19 ± 2 |
| MEA61 | 3.6 ± 0.7 | 5.5 ± 0.4 | 8 ± 0.5 | 26 ± 0,6 | 14 ± 0,8 |

To exclude major stability issues, the compound **[^99m^Tc]MEA61** was incubated with samples of human and murine blood serum (Figure 11 + Figure 12). The tracer showed no decomposition using the described procedure.


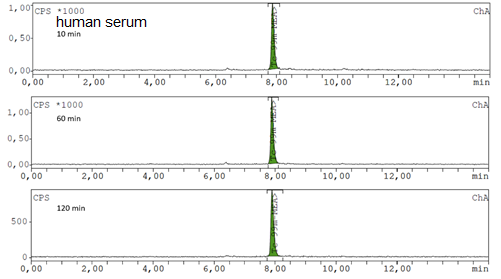


Figure 11: Evaluation of the stability of compound [^99m^Tc]MEA61 in human blood serum


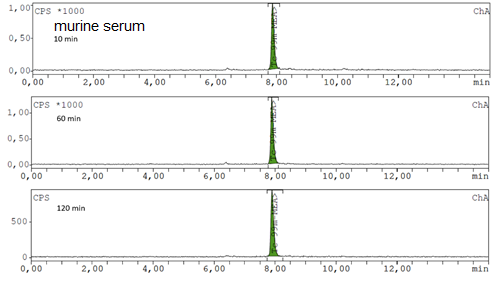


Figure 12: Evaluation of the stability of compound [^99m^Tc]MEA61 in murine blood serum

### HYNIC based barbiturate tracer [^99m^Tc]MEA223

The use of HYNIC as a ligand was published by various authors before.[19–23] The HYNIC kit was used to label 6-hydrazinyl-N-(2-{2-[2-(2-{4-[2,4,6-trioxo-5-(4-phenoxyphenyl)hexahydro-pyrimidin-5-yl]piperazin-1-yl}ethoxy)ethoxy]ethoxy}ethyl)nicotinoamid (**19**, 0.5 mg in 50 µL methanol) according to general procedure AR. The kit includes both co-ligands. The mixture was heated for 20 min and the product was isolated by radio-HPLC B (Figure 13). Used ^99m^Tc as pertechnetate: 4154 – 7167 MBq; rcy (d.c.): 34% ± 10% (n = 10); rcp: > 99%; time for synthesis: 143 min ± 30 min (n = 10); t_R_ (HPLC C) = 7.1 min.

Figure 13: Radiosynthesis of the HYNIC based barbiturate tracer [^99m^Tc]MEA223

Quality control was performed by analytical radio-HPLC C. Since am analogues Re-compound of **[^99m^Tc]MEA223** could not be synthesized, no reference was co-injected.


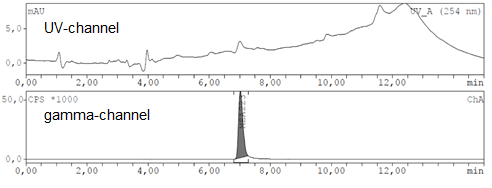


Figure 14: Quality control of the HYNIC based barbiturate tracer [^99m^Tc]MEA223

Additional quality control was performed applying mass spectrometry. The spectrum is in accordance with the expected compound (Figure 15).


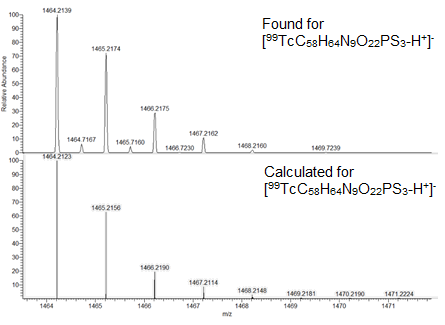


Figure 15: Orbitrap MS-spectra of compound [^99^Tc]MEA223

To exclude major stability issues, the compound **[^99m^Tc]MEA223** was incubated with samples of human and murine blood serum (Figure 16 + Figure 17). The tracer showed no decomposition using the described procedure.


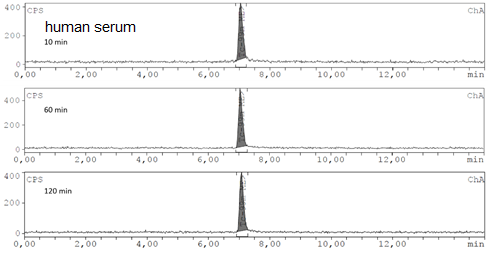


Figure 16: Evaluation of the stability of compound [^99m^Tc]MEA223 in human blood serum


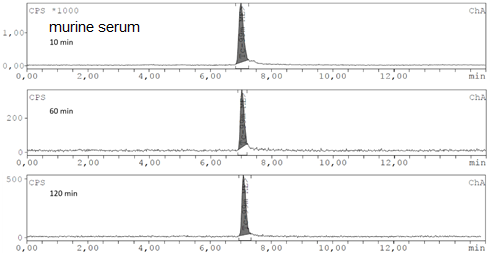


Figure 17: Evaluation of the stability of compound [^99m^Tc]MEA223 in murine blood serum

## 1.4. Western blot

To show which MMP isoforms are present in the subcutaneous thyroid tumors, we performed western blots for clinically relevant MMP subtypes MMP-2, MMP-9, and MMP-13.

For western blot analysis, K1 tumors were rapidly frozen in liquid nitrogen and ground by a Mikrodismembrator (Sartorius, Göttingen, Germany). The resulting powder was reconstituted in ice-cold NaCl buffer (50 mM Tris/HCl pH 7.5, 75 mM NaCl containing 1 mM phenylmethylsufonyl fluoride). After centrifugation (4 °C, 20 minutes, 13,000*g*), total protein amounts were measured using a protein assay kit (Bio-Rad, Munich, Germany). Ten or twenty micrograms of total protein were separated in nonreducing sodium dodecyl sulfate (SDS)-polyacrylamide gel electrophoresis (PAGE) gels. Proteins were blotted according to standard protocols with details as follows. Membranes were blocked in 3 % bovine serum albumin or milk powder in Tris-buffered saline/0.1% Tween-20 (TBST), and incubated at 4°C overnight with rabbit polyclonal anti-MMP-2 (1:1000 dilution; Santa Cruz Biotechnology, Heidelberg, Germany), rabbit polyclonal anti-MMP-9 (1:1000 dilution; abcam, Cambridge, United Kingdom), rabbit polyclonal anti-MMP-13 (1:3000 dilution; abcam, Cambridge, United Kingdom), and mouse monoclonal anti-actin (1:5000 dilution; MP Biomedicals, Eschwege, Germany). Blots were washed in TBST and incubated with a secondary goat anti-rabbit IgG-HRP antibody (1:1000 dilution; DAKO) or rabbit antimouse IgG-HRP antibody (1:10,000 dilution; DAKO) in TBST for 1 hour at room temperature. After washing with TBST, membranes were developed using the enhanced chemiluminescence detection system (Perkin Elmer, Waltham, MA) and analysed using the IVIS Spectrum imaging system (Perkin Elmer).


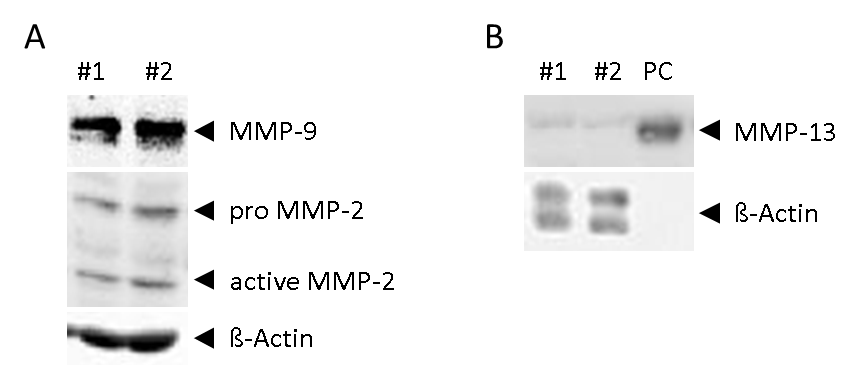


**Figure 18: Western blot analysis of thyroid tumors confirmed a high expression of MMP-9 and MMP-2 (A) and a weak expression of MMP-13 (B). PC = positive control**

# Bibliography

1. Mindt TL, Struthers H, Spingler B, et al (2010) Molecular Assembly of Multifunctional ^99m^Tc Radiopharmaceuticals Using “Clickable” Amino Acid Derivatives. ChemMedChem 5:2026–2038. https://doi.org/10.1002/cmdc.201000342

2. Faust A, Waschkau B, Waldeck J, et al (2008) Synthesis and Evaluation of a Novel Hydroxamate Based Fluorescent Photoprobe for Imaging of Matrix Metalloproteinases. Bioconjug. Chem. 19:1001–1008. https://doi.org/10.1021/bc700409j

3. Alberto R, Egli A, Abram U, et al (1994) Synthesis and reactivity of [NEt_4_]_2_[ReBr_3_(CO)_3_]. Formation and structural characterization of the clusters [NEt_4_][Re_3_(µ_3_-OH)(µ-OH)_3_(CO)_9_] and [NEt_4_][Re_2_(µ-OH)_3_(CO)_6_] by alkaline titration. J. Chem. Soc., Dalton Trans. 0:2815–2820. https://doi.org/10.1039/DT9940002815

4. Faust A, Waschkau B, Waldeck J, et al (2008) Synthesis and Evaluation of a Novel Fluorescent Photoprobe for Imaging Matrix Metalloproteinases. Bioconjug. Chem. 19:1001–1008. https://doi.org/10.1021/bc700409j

5. Mindt TL, Struthers H, Spingler B, et al (2010) Molecular Assembly of Multifunctional ^99m^Tc Radiopharmaceuticals Using “Clickable” Amino Acid Derivatives. ChemMedChem 5:2026–2038. https://doi.org/10.1002/cmdc.201000342

6. Radinov R, Hutchings S, Liu W (2006) Palladium-catalyzed Arylation of Diisopropyl Malonate Applied to the Efficient Synthesis of the Selective MMP Inhibitor 5-(4-Phenoxyphenyl)-5-[4-(2-pyrimidinyl)-1-piperazinyl]barbituric Acid. Heterocycles 67:763–768. https://doi.org/10.3987/COM-05-S(T)40

7. Hooft, R. W. W. NB v. (1998) COLLECT, Program for Collecting Data on CCD Area Detectors

8. Otwinowski Z, Minor W (1997) Processing of X-ray diffraction data collected in oscillation mode. Meth. Enzymol. 276:307–326. https://doi.org/10.1016/S0076-6879(97)76066-X

9. Otwinowski Z, Borek D, Majewski W, Minor W (2003) Multiparametric scaling of diffraction intensities. Acta Crystallogr. A 59:228–234. https://doi.org/10.1107/S0108767303005488

10. Sheldrick GM (2015) SHELXT - Integrated space-group and crystal-structure determination. Acta Crystallogr. A 71:3–8. https://doi.org/10.1107/S2053273314026370

11. Sheldrick GM (2015) Crystal structure refinement with SHELXL Acta Crystallogr. C. 71:3–8. https://doi.org/10.1107/S2053229614024218

12. Bruker AXS (1998) XP – Interactive molecular graphics

13. Prante O, Hocke C, Löber S, et al (2006) Tissue distribution of radioiodinated FAUC113 Assessment of a pyrazolo(1,5-a) pyridine based dopamine D4 receptor radioligand candidate. Nuklearmedizin 45:41–48

14. Huang W, Meng Q, Suzuki K, et al (1997) Mutational Study of the Amino-terminal Domain of Human Tissue Inhibitor of Metalloproteinases 1 (TIMP-1) Locates an Inhibitory Region for Matrix Metalloproteinases. J. Biol. Chem. 272:22086–22091. https://doi.org/10.1074/jbc.272.35.22086

15. Alberto R, Ortner K, Wheatley N, et al (2001) Synthesis and properties of boranocarbonate: a convenient in situ CO source for the aqueous preparation of [(^99m^Tc)(OH(_2_))_3_(CO)3]^+^. JACS 123:3135–6

16. Eckardt J, Geworski L, Lerch H, et al (2009) Empfehlungen zur Qualitätskontrolle in der Nuklearmedizin. Schattauer

17. Ji S, Czerwinski A, Zhou Y, et al (2013) ^99m^Tc-Galacto-RGD2: a novel ^99m^Tc-labeled cyclic RGD peptide dimer useful for tumor imaging. Mol. Pharm. 10:3304–14. https://doi.org/10.1021/mp400085d

18. Mindt TL, Struthers H, Brans L, et al (2006) “Click to chelate”: Synthesis and installation of metal chelates into biomolecules in a single step. JACS 128:15096–15097. https://doi.org/10.1021/ja066779f

19. Su H, Spinale FG, Dobrucki LW, et al (2005) Noninvasive targeted imaging of matrix metalloproteinase activation in a murine model of postinfarction remodeling. Circulation 112:3157–67. https://doi.org/10.1161/CIRCULATIONAHA.105.583021

20. Liu S (2008) Bifunctional coupling agents for radiolabeling of biomolecules and target-specific delivery of metallic radionuclides. Adv. Drug Deliv. Rev. 60:1347–1370. https://doi.org/10.1016/J.ADDR.2008.04.006

21. Purohit A, Liu S, Ellars CE, et al (2004) Pyridine-containing 6-hydrazinonicotinamide derivatives as potential bifunctional chelators for ^99m^Tc-labeling of small biomolecules. Bioconjug. Chem. 15:728–737. https://doi.org/10.1021/bc034141c

22. Purohit A, Liu S, Casebier D, Edwards DS (2003) Phosphine-containing HYNIC derivatives as potential bifunctional chelators for ^99m^Tc-labeling of small biomolecules. Bioconjug. Chem. 14:720–727. https://doi.org/10.1021/bc034059h

23. Matusiak N, Waarde A, Bischoff R, et al (2013) Probes for Non-invasive Matrix Metalloproteinase-targeted Imaging with PET and SPECT. Curr. Pharm. Des. 19:4647–4672. https://doi.org/10.2174/1381612811319250011
